# SCOUT: simultaneous time segmentation and community detection in dynamic networks

**DOI:** 10.1038/srep37557

**Published:** 2016-11-24

**Authors:** Yuriy Hulovatyy, Tijana Milenković

**Affiliations:** 1 Department of Computer Science and Engineering, Eck Institute for Global Health, and Interdisciplinary Center for Network Science and Applications (iCeNSA); University of Notre Dame, Notre Dame, IN 46556, USA

## Abstract

Many evolving complex real-world systems can be modeled via dynamic networks. An important problem in dynamic network research is community detection, which finds groups of topologically related nodes. Typically, this problem is approached by assuming either that each time point has a distinct community organization or that all time points share a single community organization. The reality likely lies between these two extremes. To find the compromise, we consider community detection in the context of the problem of segment detection, which identifies contiguous time periods with consistent network structure. Consequently, we formulate a combined problem of segment community detection (SCD), which simultaneously partitions the network into contiguous time segments with consistent community organization and finds this community organization for each segment. To solve SCD, we introduce SCOUT, an optimization framework that explicitly considers both segmentation quality and partition quality. SCOUT addresses limitations of existing methods that can be adapted to solve SCD, which consider only one of segmentation quality or partition quality. In a thorough evaluation, SCOUT outperforms the existing methods in terms of both accuracy and computational complexity. We apply SCOUT to biological network data to study human aging.

Networks (or graphs) are elegant yet powerful abstractions for studying complex systems in various domains, from biological entities to social organizations. Real-world systems evolve over time. Until relatively recently, dynamic measurements about their functioning have been unavailable, owing mostly to limitations of technologies for data collection. Hence, an evolving system has traditionally been analyzed by studying its static network representation, which discards the system’s time dimension by combining all of its interacting elements and their connections across multiple times into a single aggregate network. For example, dynamic cellular functioning has traditionally been modeled as a static protein-protein interaction (PPI) network that combines biomolecular interactions across different time points and other contexts^1^. Such an aggregate approach loses important temporal information about the functioning of evolving systems^2^. Analyzing dynamic network representations of evolving systems is crucial for understanding mechanisms behind various dynamic phenomena such as human aging in the computational biology domain^3^ or opinion formation in the social network domain^4^, especially with the increasing recent availability of temporal real-world data in these and other domains. The dynamic network representation of an evolving system models its temporal measurement data as a series of network snapshots, each of which encompasses the temporal data observed during the corresponding time interval. We refer to this snapshot-based representation as a dynamic network.

Approaches for studying dynamic networks can be categorized into: 1) those that extend well-established static network problem formulations and solutions to their dynamic counterparts, and 2) those that consider novel network problems and solutions that arise specifically from the time dimension and are thus native only to the dynamic setting. A popular problem from category 1 above that is of our interest is *community detection*. A popular problem from category 2 above that is of our interest is *time segmentation*, or *segment detection* (also known as *change detection*). We next discuss these two problems.

Community detection studies network structure (or topology) from mesoscopic (intermediate or groups-of-nodes level) perspective, in contrast to doing so from macroscopic (global or network level) or microscopic (local or node level) perspective^5^. Its goal is to identify groups of topologically related (e.g., densely interconnected^6^,^7^ or topologically similar^8^,^9^,^10^) nodes called *communities* (or *clusters*), which are likely to indicate important functional units within the network. Examples of communities are groups of proteins with similar functions in a biological network or groups of friends in a social network^5^,^11^,^12^. We focus on the mathematical notion of a *partition*, which divides a network into non-overlapping communities. Yet, our work can be extended to handle overlapping communities as well. For an evolving real-world system, community detection in its dynamic network representation is likely to yield additional insights compared to community detection in its static representation^13^,^14^. Two extremes of community detection in a dynamic network are: 1) *snapshot clustering* and 2) *consensus clustering*. On the one hand, snapshot clustering finds a separate partition for each temporal snapshot^15^,^16^,^17^,^18^,^19^. Given the snapshot-level partitions, one can then track their evolution by matching individual clusters in adjacent snapshots^20^,^21^,^22^,^23^. On the other hand, consensus clustering finds a single partition that fits well all snapshots^24^,^25^,^26^,^27^. In the real life, community organization most often lies between these two extremes. Finding this real life community organization is one of key goals of our study.

Segment detection aims to divide a dynamic network into continuous *segments* (groups of snapshots), such that the “border” between each pair of adjacent segments marks a prominent shift in the network structure^28^. As a result, all snapshots within a given segment have similar network structure, while every two adjacent segments have snapshots with dissimilar structure. The set of all segments covering the whole dynamic network is called the *segmentation* of the network. Time points that separate the segments are called *change points*. Since change points correspond to shifts in the network structure, they likely indicate functionally important events in the life of the underlying system^28^. For example, change points can correspond to transitions between different functional states in brain networks or to stock market changes in financial networks^29^. Finding change points indicating important structural shifts in the dynamic network is the other key goal of our study.

Community detection partitions a dynamic network along the node dimension (by grouping nodes into communities), while segment detection does this along the time dimension (by grouping snapshots into segments). Their combination, which is our focus and which we refer to as *segment community detection (SCD*), can be seen as two-dimensional clustering: simultaneously grouping snapshots of the dynamic network into segments based on community organization of the snapshots, and grouping nodes of the snapshots into communities based on the segments these snapshots belong to (Fig. 1(a)).

SCD naturally allows for compromising between the extremes of snapshot clustering and consensus clustering to identify the real life community organization. While snapshot clustering “zooms in” to each snapshot and consensus clustering “zooms out” to the whole network, SCD can automatically choose an appropriate “zoom level” by focusing on segments, each spanning coherent snapshots while still capturing important changes in the community organization (Fig. 1(b)). Consider studying how protein modules evolve with age: it may be more desirable to focus on different stages of aging (infancy, childhood, adolescence, adulthood, etc.^30^) via SCD than on each day/month/year of the lifespan via snapshot clustering or on the entire lifespan via consensus clustering. Similar holds when studying e.g., evolution of protein modules with disease progression.

Existing approaches that can be adapted to be able to deal with the SCD problem are GraphScope^31^, Multi-Step^25^, and GHRG^32^ (Supplementary Section S1). These methods can produce both segments and the corresponding partitions, which *is* a solution that SCD aims to find. However, they have drawbacks. **1)** They generally cannot produce a high-quality solution with respect to both SCD aspects (segmentation quality and partition quality), as we show in Results. **2)** For each method, the number of segments can only be either: a) determined automatically but not set by the user, or b) set by the user but not determined automatically. In applications where domain expert knowledge on the desired number of segments is available, the user should be able to feed this knowledge into the method by setting the number of segments, but the methods of type “a” above (GraphScope and GHRG) cannot handle this. In applications where such knowledge is unavailable, the method should be able to determine an appropriate number of segments automatically, but the methods of type “b” above (Multi-Step) cannot handle this. For a method to be generalizable to both application types, it should be able to handle both automatic and user-defined determination of the appropriate number of segments. **3)** Each of the existing methods has a single built-in intuition about what a good segment or partition is. Hence, each approach could be biased towards the particular parameters that it implements. Thus, a generalizable approach that would offer flexibility in terms of parameter choices is desirable. To address these three drawbacks, we introduce *SCOUT*, a new general framework for segment community detection, as follows.

We propose a novel SCD formulation as an optimization process that integrates the two aspects (segment detection and community detection) more explicitly than the existing methods. Also, we propose SCOUT, a general framework for solving the new formulation, which addresses the above drawbacks: **1)** it can produce a high-quality solution with respect to both SCD aspects; **2)** it can handle both automatic and user-defined determination of the appropriate number of segments; **3)** it offers high level of flexibility when it comes to the choice of segmentation or partition quality parameters.

SCOUT’s key algorithmic components (Supplementary Section S2.1 and Figures S1–S3) are: *objective function* (a measure of what a good SCD solution is), *consensus clustering* (given a set of change points, how to find a good partition for each segment), and *search strategy* (how to search through the space of possible change point sets). We vary choices for these components to trade off between different goals, such as segmentation quality and partition quality, or accuracy and speed.

We comprehensively evaluate SCOUT against the existing methods on both synthetic and real-world networks of varying sizes (Supplementary Section S2.2 and Figures S4–S5). In particular, to illustrate generalizability of our approach, we first perform evaluation on synthetic dynamic network data. For this purpose, we introduce an intuitive model for automatic generation of a synthetic dynamic network of an arbitrary size with known ground truth segmentation as well as community organization; we analyze 20 different synthetic ground truth configurations. In addition, we analyze six real-world dynamic networks from domains that do offer such data and that offer such data with some ground truth knowledge embedded into them; these networks span studies of human proximity, communication, and political relationships. To evaluate how well each method can reconstruct the ground truth knowledge, we rely on established partition quality and similarity measures as a basis for developing new SCD accuracy measures that can simultaneously account for both segmentation quality and partition quality. We show that SCOUT outperforms the existing methods with respect to both segmentation quality and partition quality, while also being more computationally efficient. In a case study, we show that SCOUT, when applied to dynamic, age-specific human PPI network data^3^,^33^, correctly identifies different stages of the aging process. We make our SCOUT implementation publicly available (http://nd.edu/~cone/SCOUT).

## Results

For each method (GraphScope, Multi-Step, GHRG, and SCOUT), we first evaluate the effect of parameter choices on its performance (Supplementary Section S3 and Figures S6–S12). Then, we compare the methods under their best parameter values on synthetic and real-world networks, via network structure-based and ground truth knowledge-based measures (see Methods). As a measure of the former type, we use average snapshot partition quality *Q*_*P*_ based on modularity. As a measure of the latter type, we use a) similarity of a method’s output to the ground truth and b) change point classification. For case “a” above, we compute segmentation similarity *Sim*_*T*_, partition similarity *Sim*_*P*_, and overall similarity *Sim*_*B*_; for all three measures, we rely on Normalized Mutual Information (NMI). For case “b” above, we use area under the precision-recall curve (AUPR).

When we have the complete ground truth SCD information (on both the segmentation aspect and the partition aspect) available, which is the case for our synthetic networks, we use all of the above measures, but we trust *Sim*_*B*_ the most, since it captures similarity between a given method’s solution and the ground truth solution with respect to both SCD aspects. When we do not have the complete ground truth information, which is the case for our real-world networks, we cannot use the two-aspect *Sim*_*B*_. Instead, we use the structure-based measure (*Q*_*P*_ based on modularity) and whichever ground truth knowledge-based measure we *can* compute based on the partial ground truth information. Since in our case the available ground truth information is the list of change points (see Methods), for the latter, we can use any measure that captures the segmentation aspect of the solution quality. We have two such measures: *Sim*_*T*_ and change point classification. Since we show below that the two measures yield consistent results on synthetic networks, and since per our discussion in Methods change point classification is theoretically more meaningful than *Sim*_*T*_ (as it accounts for ranking of all time points rather than only for the identified change points), for brevity, we focus on change point classification for real-world networks.

### Synthetic networks

We evaluate the methods on 20 synthetic network configurations: five options for the number of segments times four options for the number of nodes per snapshot; each configuration has 16 snapshots. These configurations span the whole “spectrum” between the extreme cases of snapshot clustering (where the number of ground truth segments corresponds to the number of snapshots) and consensus clustering (where there is only one ground truth segment – the whole dynamic network). For each configuration, we generate multiple random network instances and report results averaged over the multiple instances.

The idea of our synthetic network generator (snapshots within the same segment having the same community organization) aligns well with the intuition of each considered method. Thus, each method has a fair chance for recovering the ground truth knowledge, except Multi-Step, which has an *unfair advantage*. Namely, to be able to evaluate Multi-Step, we need to provide the ground truth number of segments as input to it (Supplementary Section S3). We denote this modification as Multi-Step*. This *a priori* knowledge gives an unfair advantage to Multi-Step for all configurations and especially for the extreme configurations with the minimum and maximum possible numbers of ground truth segments (i.e., with one and 16 segments, respectively). This is because for these two types of configurations, the knowledge of the ground truth number of segments guarantees that Multi-Step’s solution will have the correct segmentation: given all 16 snapshots, there is only one way to group the 16 snapshots into one segment (the resulting segment will encompass all 16 snapshots) and only one way to group the 16 snapshots into 16 segments (each segment will encompass exactly one snapshot). For the other non-extreme configurations, while knowing the ground truth number of segments still gives an advantage to Multi-Step (as it will produce the correct ground truth number of segments, or equivalently, the correct number of change points), it does not necessarily guarantee that Multi-Step will obtain the correct segmentation (i.e., that the identified change points will be correct). This is because for the non-extreme configurations, there are multiple ways to group snapshots into the given number of segments.

Below, we discuss results for the segmentation aspect (*Sim*_*T*_ and change point classification), partition aspect (*Q*_*P*_ and *Sim*_*P*_), and both aspects (*Sim*_*B*_) of SCD. For *Sim*_*B*_, since we trust it the most, we test the statistical significance of SCOUT’s improvement over the existing methods (Supplementary Section S2.2). Finally, we compare the methods’ running times.

#### Segmentation aspect of the solution quality

For *Sim*_*T*_, SCOUT is superior to all other methods, as it achieves the highest scores for 90% of all synthetic network configurations, while the other methods are comparable to each other (Supplementary S13). The remaining 10% (i.e., two) of all configurations in which an existing method (in this case, GraphScope) achieves higher scores are configurations with the two largest numbers of nodes per snapshot and with the maximum possible number of segments (Supplementary S14). The fact that GraphScope has higher *Sim*_*T*_ for these configurations is not surprising. Namely, GraphScope produces solutions with more segments than the other methods do, often overestimating the ground truth number of segments (Supplementary S15). So, for the configurations with the maximum possible number of segments, the most that GraphScope can overestimate is the maximum number of segments itself, i.e., the correct solution. Note that when measuring *Sim*_*T*_ for the extreme configurations with the minimum and maximum possible numbers of segments, we exclude Multi-Step from comparison. This is because of Multi-Step’s unfair advantage (see above), which for these extreme configurations means *a priori* knowing the correct segmentation and thus achieving the perfect *Sim*_*T*_ (Supplementary S14). For the remaining non-extreme configurations, Multi-Step is always outperformed by SCOUT and at least one of the existing methods (Supplementary S13). Thus, Multi-Step, which knows the ground truth number of segments *a priori* typically does not yield a high quality segmentation with respect to *Sim*_*T*_, whereas SCOUT does so (and typically better than the other methods) despite not having this prior knowledge (Fig. 2(a) and Supplementary S14). This is further confirmed by SCOUT being able to automatically determine the ground truth number of segments more accurately than the existing methods (Supplementary S15).

For change point classification, the trends are consistent to those for *Sim*_*T*_: SCOUT is again superior in over 90% of all synthetic network configurations (Fig. 2(b) and Supplementary Figures S13 and S16). The consistency is expected, since the two measures focus on the same aspect of SCD. Note that unlike for *Sim*_*T*_ above, for change point classification, Multi-Step does not have the unfair advantage over the other methods (Supplementary Section S2.2).

#### Partition aspect of the solution quality

For *Q*_*P*_, SCOUT is superior to all other methods, achieving the highest *Q*_*P*_ for 70% of all synthetic network configurations (Supplementary S17). Of the existing methods, Multi-Step is the best, followed by GraphScope and GHRG that are comparable to each other (Fig. 2(c) and Supplementary S16). Importantly, SCOUT overall outperforms Multi-Step in terms of *Q*_*P*_ despite the fact that Multi-Step explicitly maximizes modularity (which is the basis of *Q*_*P*_), while the version of SCOUT under consideration does not rely on *Q*_*P*_ (Supplementary Section S3). The configurations on which Multi-Step outperforms SCOUT are mostly those with the maximum possible number of ground truth segments (Fig. 2(c) and Supplementary S16). This is not surprising, since for these 16-segment configurations, SCOUT can produce a solution with *at most* 16 segments, while Multi-Step is guaranteed to produce the solution with exactly 16 segments. That is, intuitively, Multi-Step’s solution has a separate segment partition for each snapshot, and each of the partitions aims to maximize modularity and consequently *Q*_*P*_. For the configurations where Multi-Step outperforms SCOUT, Multi-Step’s *Q*_*P*_-based superiority is *not* necessarily an advantage. This is because Multi-Step achieves higher *Q*_*P*_ scores even compared to *Q*_*P*_ scores of the ground truth solution (Fig. 2(c) and Supplementary S16). Thus, even if Multi-Step obtains the highest *Q*_*P*_, its partitions might not necessarily be closer to the ground truth than SCOUT’s partitions, as we justify next.

For *Sim*_*P*_, SCOUT is superior to all other methods, achieving the highest *Sim*_*P*_ score for all configurations (Supplementary S17). The other methods are relatively comparable to each other, with slight superiority of Multi-Step (Fig. 2(a) and Supplementary S14). Trends for *Sim*_*P*_ are not always consistent with those for *Q*_*P*_, although the two capture the same SCD aspect. For example, for the configuration with 100 nodes per snapshot and 16 ground truth segments, although Multi-Step achieves the highest *Q*_*P*_ score (Fig. 2(c)), it has the lowest *Sim*_*P*_ score (Supplementary S14). The inconsistency between *Q*_*P*_ and *Sim*_*P*_ is not be surprising, since modularity is not always able to capture well the ground truth communities^7^.

#### Overall solution quality

For *Sim*_*B*_, SCOUT again outperforms the other methods for all configurations (Fig. 3). The other methods are comparable to each other (Fig. 2(d) and Supplementary S16). Importantly, in most cases, SCOUT not only improves upon the existing methods but also its improvement is statistically significant. Namely, SCOUT statistically significantly improves upon the best existing method in 75%, 65%, and 55% of all cases at *p*-value threshold of 0.05, 0.01, and 0.001, respectively (Supplementary Table S1). Note that the above percentages could not be perfect, since for 20% of all configurations (namely, the four configurations with the minimum number of ground truth segments), in addition to SCOUT that achieves the perfect *Sim*_*B*_, Multi-Step also (unfairly, per our above discussion) achieves the perfect *Sim*_*B*_ and is thus comparable to SCOUT.

#### Running time

Theoretic computational complexities of all existing methods and SCOUT are discussed in Supplementary Sections S1 and S2.1, respectively. Here, we focus on comparing the methods’ empirical running times. SCOUT is the fastest of all methods, over all configurations (Supplementary S18). Specifically, across all network sizes, SCOUT is at least 2.6 times faster than the next fastest method. It is followed by Multi-Step, GraphScope, and GHRG, respectively. GHRG, even when parallelized, cannot be run for the larger networks due to its high computational complexity.

### Real-world networks

We evaluate all methods on six real-world networks. Since the complete ground truth knowledge (both change points and segment partitions) is unavailable for any of the networks, we perform evaluation based on change point classification and *Q*_*P*_.

#### Segmentation aspect of the solution quality

For change point classification, SCOUT is superior to all other methods, achieving the highest accuracy for all considered real-world networks, and it is followed by GHRG, GraphScope, and Multi-Step, respectively (Fig. 4(a)). This method ranking is consistent with that for synthetic networks. We have formal lists of change points only for four of the six networks, and thus the above change point classification is performed only on those four networks. Yet, we can still intuitively (informally) analyze segmentation results of the methods for the remaining two networks, *High School* and *Senate*, as follows.

Regarding *High School* network, this network captures proximity of students in a high school. Intuitively, we do not expect large-scale changes in the students’ interaction patterns over time (meaning that we expect very few change points, if any, i.e., very few segments, possibly only one), since students typically interact with other students from the same classes^34^. Consistent with this intuition, SCOUT (as well as GraphScope and Multi-Step) detects only one segment for *High School* network (Supplementary S19). Moreover, SCOUT (as well as Multi-Step) produces the partition for this single segment that perfectly matches the (static) partition of students according to their classes^34^. Hence, it is encouraging that SCOUT (as well as Multi-Step) captures the intuition about the expected dynamics and structure of *High School* network.

Regarding *Senate* network, for a given method, we identify its 10 top-ranked “change point”-like time points. The lists of top ranked time points produced by the different methods have little overlap (Supplementary S20). Specifically, the four methods combined identify 33 out of all 4 × 10 = 40 possible distinct time points. Also, there is only one time point (the 83^*rd*^ Congress in 1953) that is identified by more than two methods (SCOUT, GraphScope, and GHRG). Clearly, the results of the different methods are quite complementary. We aim to empirically evaluate whether the top ranked points correspond to important historical events. If so, this would further validate the given method. This evaluation needs to be performed qualitatively (rather than quantitatively, as done so far), since it is hard to determine the ranking of all historical events in terms of their importance and consequently to correlate this ranking with the methods’ ranking of the time points. Because of this, and because the resulting qualitative evaluation is time consuming, while we illustrate the top 10 ranked change points for each method (Supplementary S20), here we do not compare the different methods. Instead, we discuss SCOUT’s results only, to at least intuitively assess their meaningfulness. SCOUT’s top four time points (1953, 1879, 2003, and 1979, respectively) correspond to Congresses with shifts in the structure of the Senate’s majority between the Democratic and Republican parties. Of SCOUT’s next three time points (the 86^*th*^, 88^*th*^, and 67^*th*^ Congress, respectively), the first two brought major civil rights acts (Civil Rights Act of 1960 and Civil Rights Act of 1964, respectively), and during the third one, “Teapot Dome” Scandal occurred, which is considered one of the most significant investigations in the history of the Senate^35^. SCOUT’s remaining three time points correspond to divided Congresses: the 112^*th*^ Congress that almost lead to government shutdown^36^, plus the 80^*th*^ Congress and the 109^*th*^ Congress, both of which were nicknamed as “do-nothing”^37^. It is encouraging that SCOUT identifies as likely change points such important historical events.

#### Partition aspect of the solution quality

For *Q*_*P*_, except for *Hypertext* and *AMD Hope* networks, SCOUT and Multi-Step are comparable, and they outperform GraphScope and GHRG (Fig. 4(b)); this is the same trend as for synthetic networks. For *Hypertext* network, SCOUT is outperformed by GHRG and Multi-Step, respectively (Fig. 4(b)). For *AMD Hope* network, SCOUT is outperformed by Multi-Step and GraphScope, respectively (Fig. 4(b)). These results for *Hypertext* and *AMD Hope* networks are not necessarily surprising. Namely, different methods can produce solutions with different numbers of segments. In particular, for these two networks, GHRG and GraphScope produce more segments than SCOUT and Multi-Step (Supplementary S19). The more segments exist in a solution, the easier it is for this solution to obtain a high partition quality score (i.e., *Q*_*P*_). Hence, a direct comparison of *Q*_*P*_ scores of the solutions with different numbers of segments may be inappropriate. Consider this: if method 1 has a slightly higher *Q*_*P*_ score than method 2, but it also achieves its score with ten times as many segments as method 2, does it mean that method 1 has a better partition accuracy than method 2? Probably not. Thus, ideally, we want to compare *Q*_*P*_ scores of the solutions with equal numbers of segments. Since SCOUT can produce a solution with not only an automatically determined but also user-provided number of segments, we can compare *Q*_*P*_ score of each existing method and *Q*_*P*_ score of SCOUT when SCOUT is asked to produce a solution with the same number of segments as the solution of the given existing method. This way, we avoid the bias arising from the fact that the two compared methods might have different numbers of segments. According to this evaluation, SCOUT outperforms all methods (Fig. 4(c) and Supplementary S21).

The shape of the *Q*_*P*_-curve as a function of the number of segments *l* from e.g., 4(c) could provide insights into the dynamics of the given network. Although the *x*-axis of the curve does not correspond to time, and thus it cannot say when changes in community organization (if any) occur, the fact that the *x*-axis corresponds to *l* can say something about the number of such changes and their scale. Namely, if *Q*_*P*_ increases slowly (or does not increase) as *l* increases, this could mean that the community organization does not change a lot with increase in the number of segments, and thus, the increase in the number of segments in unnecessary. This is the case for e.g., *High school* network (Supplementary S21), which agrees with our discussion above. On the other hand, if *Q*_*P*_ increases drastically as *l* increases, this could mean that the community organization indeed changes a lot with increase in the number of segments, and thus, the increase in the number of segments is justified. This is the case for e.g., *Senate* network (Fig. 4(c)), which also agrees with our discussion above.

#### Running time

When we compare the methods’ empirical running times, just as for synthetic networks, SCOUT is the fastest of all methods, over all real-world networks (Fig. 4(d)). Again, GHRG is the slowest method, which means that it cannot be run for the larger networks due to its high computational complexity (Supplementary Section S1).

### Case study: using SCOUT on biological network data in the context of studying human aging

Studying human aging is important, because the risk of disease increases with age. Studying human aging experimentally is hard due to long lifespan and ethical constraints. Thus, human aging has typically been studied computationally, via genomic sequence or gene expression analyses^3^. Recently, we used network analysis to deepen our knowledge about human aging. Specifically, we inferred dynamic, age-specific PPI network data of human, capturing which proteins interact at which age, and analyzed the resulting network data to identify key players in aging, i.e., proteins whose PPI network positions significantly change with age^3^. Here, we analyze the age-specific PPI network data with a different goal: to test whether SCOUT’s top ranked time points will correspond to ground truth ages that reflect shifts from one stage of human lifespan to another.

Specifically, in the age-specific PPI network data, each network corresponds to a certain age, and there are 37 networks (i.e., shapshots) for ages between 20 and 99^3^. Since we do not have available the ground truth knowledge for ages above 80 (see below), we focus only on ages between 20 and 80. We apply SCOUT, the best of all considered methods, on the resulting 23 age-specific snapshots (*x*-axis of Fig. 5). We study SCOUT’s top ranked time points (i.e., ages) to evaluate their meaningfulness. In particular, we test whether SCOUT’s top ranked ages correspond to ground truth borders between known aging stages. In our considered age range between 20 and 80 years, there are two such ground truth borders: 35 years, the change from early adulthood to midlife, and 50 years, the change from midlife to mature adulthood (vertical red lines in Fig. 5)^30^. Since the data does not contain snapshots corresponding to these two ages, we consider the next closest ages that are present in the data. For age of 50, the closest ages present in the data are 48 and 52 (Fig. 5). For age 35, we also consider ages two years before and after, i.e., ages 33 and 37 (both of which are present in the data). Moreover, it is known that an important aging-related change happens between 60 and 80 years, though it is not known exactly when^38^.

Given the three resulting ground truth age intervals (33–37, 48–52, and 60–80; gray rectangles in Fig. 5), since we expect one major change within each interval, we consider SCOUT’s top three ranked ages, hoping that each would fall within one of the intervals. SCOUT’s output are ages 37, 52, and 69 (Fig. 5). Indeed, they cover the three intervals (*p*-value of 0.03162; Supplementary Section S2.2). It is encouraging that SCOUT can perfectly recover the expected aging-related shifts.

## Discussion

We combine community detection with segment detection in dynamic networks to formulate a new problem of SCD. To address the drawbacks of the existing methods that can be employed to solve the SCD problem, we introduce a new and superior approach called SCOUT. We show that the SCD problem and SCOUT in particular is a useful framework for studying community organization of dynamic networks, as it can identify both when communities evolve by identifying change points and how communities look like at each stage of their evolution by identifying segment partitions. The solution of the SCD problem provides a concise yet informative description of the dynamic network from the perspective of its community organization.

Our work has potential future extensions. Methodologically, SCOUT could be extended to different problem settings, such as dealing with weighted networks or overlapping communities. Application-wise, an important problem in dynamic network analysis is to choose a meaningful time scale for defining network snapshots. Usually, the time scale is chosen so that each snapshot is assumed to have the same duration, and the duration is determined empirically to fit the given application. Instead, the output of SCD could provide a systematic way for defining snapshots. Namely, the smallest meaningful traditional empirical equal-length snapshots would be used to define the initial dynamic network. Then, this network would be given as input to SCOUT to group the small snapshots with consistent community organization into larger segments. Finally, the time interval of each segment would correspond to a new, more meaningful snapshot, and collection of all such new snapshots would form a new, more meaningful dynamic network. This way, each snapshot of the new network would capture the period during which community organization is consistent. Also, the duration of different snapshots could be different. These newly constructed snapshots could then be used as input to various methods for dynamic network analysis, which could improve the quality of results compared to using the same methods on the traditionally determined empirical same-length snapshots.

This paper focuses on the problem of SCD in dynamic networks. Other important network science problems exist, such as network comparison/alignment^39^,^40^,^41^, link prediction^42^, information spreading^43^, graphlet (subgraph) mining^10^, etc. Most of these problems have already been extended from the static to dynamic network context. For example, while in a static network, link prediction is used to de-noise the network by identifying missing and spurious links^44^, in a dynamic network, its goal is to predict interactions at time *t* based on network information up to time *t* − 1^42^. Also, while graphlets have traditionally been used to characterize the structure of a static network, they have been extended into dynamic graphlets to allow for analyzing temporal data^10^. Similarly, traditional static network alignment has been extended to its dynamic counterpart^45^. Clearly, the field of dynamic network analysis, and thus our SCOUT approach that contributes to the SCD problem within this field, will only continue to gain importance with the increase in availability of temporal real-world network data.

## Methods

### Problem formulation

A *dynamic network D* is a sequence of *k* snapshots {*G*_0_, *G*_1_, …, *G*_*k*−1_}, where each snapshot *G*_*i*_ = (*V*_*i*_, *E*_*i*_) is a static graph capturing network structure during time interval *i*. A sequence of consecutive snapshots can be grouped into a segment. Formally, a *segment s* is a sequence of consecutive snapshots {*G*_*i*_, *G*_*i*+1_, …, *G*_*j*_}, *i* ≤ *j*, with *i* being its *start time, j* being its *end time*, and *j* − *i* + 1 being its *length*. A sequence of non-overlapping segments (meaning that each segment in the sequence starts right after the previous one ends) that covers the whole dynamic network (meaning that the first segment in the sequence starts at time 0 and the last segment in the sequence ends at time *k* − 1) forms a segmentation of this network. Formally, a *segmentation S* is a sequence of *l* adjacent segments {*s*_0_, *s*_1_, …, *s*_*l*−1_} such that *s*_0_ starts at time 0 and *s*_*l*−1_ ends at time *k* − 1. We can specify such a segmentation via a set *T* = {*t*_1_, *t*_2_, …, *t*_*l*−1_} of *l* − 1 time points called *change points*, such that *t*_*i*_ is the start time of segment *s*_*i*_, *i* ∈ [0, *l* − 1] (by convention, we always assume that *t*_0_ = 0).

**Figure 1:**
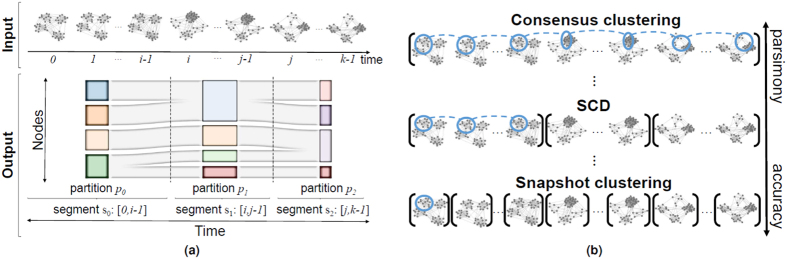
Illustrations of (**a**) our SCD problem setting and (**b**) how SCD naturally allows for compromising between the two extremes of snapshot clustering and consensus clustering. In panel (b), at each of the three horizontal levels, in blue we show the same community across different snapshots within the given segment.

**Figure 2:**
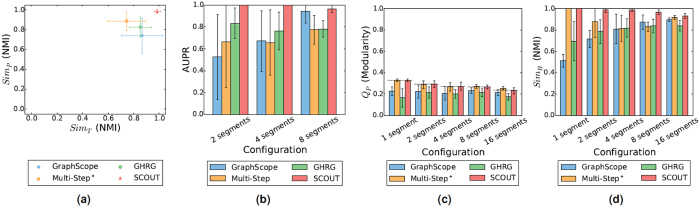
Method comparison for synthetic networks with 100 nodes per snapshot with respect to (**a**) *Sim*_*T*_ and *Sim*_*P*_ (for the configuration with four ground truth segments), (**b**) change point classification, (**c**) *Q*_*P*_, and **(d**) *Sim*_*B*_. For a given synthetic network configuration, the results are averaged overall all of the corresponding random network instances. In panel (b), we do not consider configurations with the minimum and maximum possible numbers of ground truth segments, because for these configurations, either there are no change points at all (for one segment) or every time point is a change point (for 16 segments), and thus change point classification cannot be performed. In panel (c), the dotted lines correspond to the ground truth scores. Equivalent results for the remaining configurations are shown in Supplementary Figures S14 and S16.

**Figure 3:**
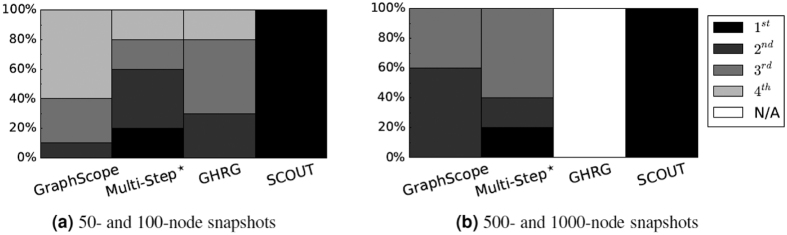
Method rankings with respect to *Sim*_*B*_ for synthetic network configurations with (**a**) 50–100 and (**b**) 500–1000 nodes per snapshot. For each configuration, we compare the four methods’ *Sim*_*B*_ scores (averages over all instances of the given configuration) to identify the first, second, third, and fourth best method; ties are allowed. We summarize these results over all considered configurations by measuring, how many times the given method (*x*-axis) is ranked as the first, second, third, and fourth best method (expressed as the percentage of all considered configurations; *y*-axis). “N/A” means that the given method (GHRG for the larger networks) could not be run. The darker the given bar, the better the method performance.

**Figure 4:**
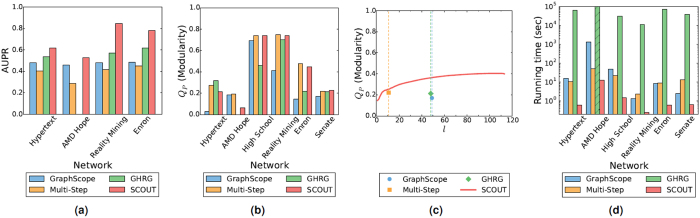
Method comparison for real-world networks with respect to (**a**) change point classification, (**b**) *Q*_*P*_, (**c**) *Q*_*P*_ (for different numbers of segments *l* for *Senate* network), and (**d**) running time (logarithmic scale). In panel (a), only networks with known ground truth change points are shown, since otherwise change point classification cannot be computed. In panel (c), *Q*_*P*_ scores of 1) SCOUT’s solutions for different numbers of segments *l* and 2) the solutions of the existing methods are shown. Specifically, for SCOUT, the line shows its *Q*_*P*_ score when solving the *Q*_*P*_-based constrained segment community detection (CSCD) problem while varying the number of segments. For each of the existing methods, the mark shows *Q*_*P*_ score of the given method’s solution, with its position along the *x*-axis corresponding to the number of segments *l* in the solution. Equivalent results to those in panel (c) for the remaining networks are shown in Supplementary S21. In panel (d), the striped bar indicates that GHRG could not be run on *AMD Hope* network due to its high computational complexity.

**Figure 5:**
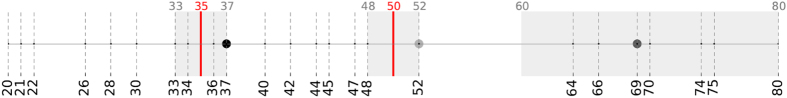
SCOUT’s top three ranked ages (circles) for the dynamic, age-specific human PPI network data. The darker the circle color, the higher the age rank. The vertical red lines and grey rectangles are explained in the text. A good approach would place each of its top three ranked ages in one of the three gray rectangles, which is exactly what SCOUT achieves.

Given a dynamic network *D*, SCD aims to simultaneously find a segmentation 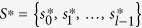 (or equivalently a change point set 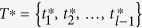) and a sequence of partitions 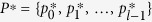 such that 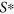 identifies important shifts in the community organization of *D* and each 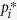 (called *segment partition*) reflects well the community organization of each snapshot within segment 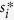 (Fig. 1(a)). The output (i.e., solution or answer) of SCD can be represented as 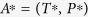. Intuitively, in a good output, 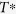 should be *parsimonious* (meaning that it should capture all important shifts in the network with as few change points as possible), while 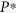 should be *accurate* (meaning that segment partitions should correctly capture community organization of all snapshots within the corresponding segment). That is, output 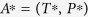 should aim to simultaneously satisfy two objectives: *segmentation parsimony* and *partition accuracy*. We can now state the problem:

### Problem 1 (SCD)

*Given a dynamic network D* = {*G*_1_, *G*_2_, …, *G*_*k*_}*, find a number of segments l, a sequence of l* − 1 *change points* 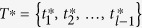
*, and a sequence of l segment partitions* 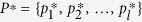 *such that the output* 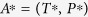 *forms a parsimonious segmentation with accurate segment partitions.*

The two objectives, segmentation parsimony and partition accuracy, are competing with each other, as optimizing one does not necessarily lead to optimizing the other. For example, at the extreme of snapshot community detection (bottom of Fig. 1(b)), each snapshot is a separate segment that has its own well-fitting partition, which yields high partition accuracy. However, such a fine-grained output with the maximum possible number of segments might contain redundancies, because some adjacent snapshots might have similar community organizations. In this case, segmentation parsimony will be low. To optimize (increase) segmentation parsimony, adjacent snapshots with similar community organizations should be grouped together. At the other extreme of consensus community detection (top of Fig. 1(b)), all snapshots form one segment with a single segment partition for the whole network, which yields high segmentation parsimony. However, the single segment partition will have to “compromise” between many possibly quite distinct snapshots. In this case, the segment partition will not be able to fit well all of the distinct snapshots, and consequently, partition accuracy will be low. In real life, the SCD solution typically lies between these two extremes, and finding it requires balancing between the two somewhat contradicting goals of optimizing both segment parsimony and partition accuracy. We formalize the ways of finding such a solution below.

Recall from Introduction the need of being able to find a solution with a user-specified number of segments *l*, in addition to being able to determine this parameter *l* automatically. Our current SCD problem formulation (Problem 1) can handle the latter scenario, but we can extend it to handle the former scenario as well. Specifically, when finding an SCD solution, in addition to allowing for simultaneously optimizing both aspects of SCD quality (segmentation parsimony and partition accuracy), we can allow for optimizing only one aspect (partition accuracy) while setting the other one (segmentation parsimony, expressed as the number of segments *l*) as a constraint. So, we extend the problem formulation by adding to the existing SCD objective from Problem 1 the following new objective: given a dynamic network *D* and the desired number of segments *l* as input by the user, find an output 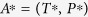 with *l* segments that achieves the highest partition accuracy. We refer to this new objective as the *constrained SCD problem (CSCD*). We propose SCOUT to solve any of the SCD and CSCD problems, to allow for handling both of the above scenarios (automatic vs. user-defined selection of the number of segments *l*, respectively).

### Our SCOUT approach

Given a dynamic network *D*, we aim to find an output 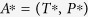 by directly optimizing an *objective function* that measures both segmentation parsimony and partition accuracy (see below for details on how we deal with SCD versus CSCD). Supplementary Algorithm S1 provides a high-level overview of SCOUT and Supplementary Section S2.1 provides further details. SCOUT has the following five steps. **1)** Select the initial change point set as the current change point set *T* (line 2 in Supplementary Algorithm S1). For example, the initial change point set could correspond to a set of all possible snapshot-level segments (bottom-up search) or just one large network-level segment (top-down search). Given *T*, the method iteratively performs the following steps. **2)** Perform *consensus clustering* within each segment *s*_*i*_ to get its corresponding partition *p*_*i*_ (line 7). In general, the consensus clustering method should aim to obtain the partition set *P* that maximizes the objective function for *T*. Step 2 results in *A* = (*T, P*) (line 9). **3)** Use a *search strategy* to search for the next change point set that will become the new current change point set *T* (line 11). Clearly, the search strategy guides how we explore the space of possible change point sets. For example, in bottom-up search, the next change point set is obtained by merging two adjacent segments, while in top-down search, the next change point set is obtained by splitting a segment into two. **4)** Repeat steps 2 and 3 above until the exploration of the space is finished (corresponding to *T* = ∅ in line 3), e.g., until one largest possible network-level segment is reached in bottom-up search or until all possible snapshot-level segments are reached in top-down search. **5)** Choose the best output out of all outputs computed in step 2 as the final output 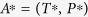 (line 13). When solving the SCD problem, the best output is the one maximizing the objective function. When solving the CSCD problem, the best output is the one maximizing the objective function while satisfying the constraint (the solution consisting of *l* segments).

### Experimental setup

#### Methods for comparison

We compare SCOUT with GraphScope, Multi-Step, and GHRG (Supplementary Section S2.2).

#### Datasets

We evaluate the methods on two types of networks: *synthetic networks* and *real-world networks*.

##### Synthetic networks

To generate a synthetic dynamic network *D* with the embedded ground truth *A*^(*gt*)^ = (*T*^(*gt*)^, *P*^(*gt*)^), we introduce a new dynamic random graph model, called *segment community generator* (SCG). Intuitively, we first select change points *T*^(*gt*)^ and generate segment partitions *P*^(*gt*)^. Then, we use these to generate *D*, assuming that each of its snapshots is generated via a stochastic blockmodel based on the corresponding segment partition. For details, see Supplementary Section S2.2 and Algorithm S2. For our experiments, we generate synthetic dynamic networks with 16 snapshots and 1, 2, 4, 8, and 16 ground truth segments, while considering various network sizes: 50, 100, 500, and 1000 nodes in each snapshot. This results in 5 × 4 = 20 synthetic network configurations. For each configuration, we generate 10 instances in order to account for the randomness in the synthetic network generator. This totals to 20 × 10 = 200 synthetic networks.

##### Real-world networks

We consider six publicly available real-world dynamic networks (Supplementary Section S2.2 and Table S2). **1)** *Hypertext*^46^ network captures face-to-face proximity of attendees of the Hypertext 2009 conference. This network has *T*^(*gt*)^ that corresponds to the list of events from the conference program^46^. **2)** *AMD Hope*^47^ network captures co-location of attendees of The Last HOPE 2008 conference. This network has *T*^(*gt*)^ that corresponds to featured/keynote talks and social events^47^. **3)** *High School*^34^ network captures proximity of students in a high school during a work week in 2013. This network does not have *T*^(*gt*)^. **4)** *Reality Mining*^48^ network captures social interactions of university students and faculty during 2004–2005 academic year. This network has *T*^(*gt*)^ that corresponds to the list of events from the academic calendar^32^. **5)** *Enron*^49^ network captures email communication of Enron employees during the 2000–2002 period. This network has *T*^(*gt*)^ that corresponds to the list of company-related events from the news^32^. **6)** *Senate*^18^ network captures voting similarities of US senators during the 1789–2015 period (i.e., for 113 Congresses). This network does not have *T*^(*gt*)^.

#### Evaluation measures

We evaluate the methods via *network structure-based* and *ground truth knowledge-based* measures.

##### Network structure-based measures

Here, we evaluate a given method’s solution 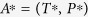 with respect to the structure of the input dynamic network *D*, without relying on any ground truth knowledge. For this, we can use any objective function from Supplementary Section S2.1. This includes four *Q*_*P*_ measures of partition quality and two *Q*_*B*_ measures accounting for both segmentation quality and partition quality. Regarding the four *Q*_*P*_ measures (modularity, conductance, normalized cut, and average-ODF), in our experiments, all four measures show statistically significantly correlated results with respect to both Pearson and Spearman correlations (with all pairwise *p*-values < 10^−49^). So, in case of *Q*_*P*_, for brevity, we report results only for modularity. Regarding the two *Q*_*B*_ measures (AIC and BIC), we do not evaluate the results with respect to them, since these are the objective functions that SCOUT explicitly optimizes, and we want to avoid circular reasoning.

##### Ground truth knowledge-based measures

Here, we explicitly rely on the ground truth knowledge to evaluate a given method’s solution 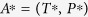, by: I) measuring similarity of 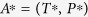 to the known ground truth solution *A*^(*gt*)^ = (*T*^(*gt*)^, *P*^(*gt*)^) and II) evaluating the method’s ability to rank time points according to how “change point-like” they are.

I) We introduce three measures of similarity between 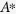 and *A*^(*gt*)^: a) *segmentation similarity Sim*_*T*_, capturing the segmentation aspect of 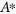 and *A*^(*gt*)^, b) *partition similarity Sim*_*P*_, capturing the partition aspect of 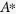 and *A*^(*gt*)^, and c) *overall similarity Sim*_*B*_, capturing simultaneously both aspects of 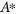 and *A*^(*gt*)^ (Supplementary Section S2.2). All of *Sim*_*T*_, *Sim*_*P*_, and *Sim*_*B*_ rely on a measure *H* of similarity between two partitions. We test four such measures *H*: **1)** *Normalized Mutual Information (NMI*)^50^, **2)** *Adjusted Mutual Information (AMI*)^50^, **3)** *Adjusted Rand Index (ARI*)^50^, and **4)** *V-Measure (VM*)^51^ (Supplementary Section S2.2). In our experiments, all four measures *H* show statistically significantly correlated results with respect to both Pearson and Spearman correlations (with all pairwise *p*-values < 10^−239^). For brevity, we report results only for NMI.

II) One way to assess a method’s ability to detect ground truth change points *T*^(*gt*)^ is via *Sim*_*T*_ from above, which directly compares the method’s change point set 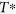 against *T*^(*gt*)^. *Sim*_*T*_ only accounts for time points that were chosen as change points, i.e., it does not consider time points that were not chosen as change points, even though some of these time points may have still been good change point candidates. Namely, when determining which time points should be change points, a method assigns to each time point a score (rank) according to how “change point-like” the time point is. Instead of using “binary” information for each time point *t* as *Sim*_*T*_ does (i.e., either *t* ∈ *T*^(*gt*)^ or 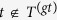), we can make use of the more complete information on ranking of all time points. This is useful because even if some ground truth change point *t* ∈ *T*^(*gt*)^ is not (mistakenly) included into 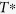, we still want the method to rank *t* higher than some other 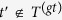. *Sim*_*T*_ would fail to capture this, so we use an alternative evaluation metric, *change point classification*, as follows. Having a ranked list of all time points (for details on how we obtain this list for each method, see Supplementary Section S2.2), we measure a given method’s performance with respect to change point classification via three measures: 1) the *area under the precision-recall curve (AUPR*), 2) the *maximum F-score*, and 3) the *area under the receiver operator characteristic curve (AUROC*) (Supplementary Section S2.2). In our experiments, all three measures show statistically significantly correlated results with respect to both Pearson and Spearman correlations (with all pairwise *p*-values < 10^−64^). So, for brevity, we report results only for AUPR.

## Additional Information

**How to cite this article**: Hulovatyy, Y. and Milenković, T. SCOUT: simultaneous time segmentation and community detection in dynamic networks. *Sci. Rep.* **6**, 37557; doi: 10.1038/srep37557 (2016).

**Publisher's note:** Springer Nature remains neutral with regard to jurisdictional claims in published maps and institutional affiliations.

## Supplementary Material

Supplementary Information
