# Supplementary material for: SCOUT: simultaneous time segmentation and community detection in dynamic networks
Source: Sci Rep. 2016 Nov 24;6:37557. doi: 10.1038/srep37557 (PMC5121586; doi:10.1038/srep37557)
Supplement: Supplementary Information [file srep37557-s1.pdf]

Supplementary information for:

SCOUT: simultaneous time segmentation and community detection in dynamic networks

Yuriy Hulovatyy and Tijana Milenković*

Department of Computer Science and Engineering
Interdisciplinary Center for Network Science and Applications (iCeNSA)
Eck Institute for Global Health
University of Notre Dame, Notre Dame, IN 46556, USA
{yhulovat, tmilenko}@nd.edu

S1 Related work

Here, we expand our discussion from the main paper on the three existing methods that can deal with the SCD problem, GraphScope, Multi-Step, and GHRG.

GraphScope [1, 2, 3] works as follows. The first snapshot becomes the current segment. Given the current segment, the method iteratively examines the next snapshot in the temporal sequence to determine whether: 1) the community organization of the snapshot in question matches well the community organization of the current segment, and thus, the snapshot should be added to the current segment (this simply extends the current segment for the next iteration), or instead 2) the community organization of the snapshot does not match well the community organization of the current segment, and thus, the snapshot should begin a new segment (which becomes the current segment for the next iteration). Community organizations of the given snapshot and the current segment are obtained and their match is measured via the minimum description length (MDL) principle. Computational complexity of the method is unknown [1].

Multi-Step [4] uses an agglomerative hierarchical clustering approach as follows. Each snapshot starts as a singleton segment. Then, in every iteration, the most similar (in terms of community organization) pair of segments are combined. Specifically, the level of similarity between two segments quantifies how well the community organization (i.e., the partition) of the first segment fits the second segment, and also how well the partition of the second segment fits the first segment. Here, the quality of the fit of a partition to a segment is based on average modularity (Supplementary Section S2.1.1), and a partition for the given segment is detected by greedily maximizing average modularity via a modification of Louvain algorithm for static community detection (Supplementary Section S2.1.2). The output of the above iterative Multi-Step procedure is a hierarchical tree with snapshots as leaves. However, it is not clear how to automatically cut the tree to obtain segments and their corresponding change points. As such, Multi-Step is suitable when the desired number of segments is provided as input. Computational complexity of the method is unknown; however, it is know that the number of required consensus clustering runs is $O(k)$, where k is the number of snapshots [4].

GHRG [5] considers a fixed-length sliding window of the most recent snapshots and uses a statistical test to evaluate whether: 1) within the window, the snapshots before and after a given time point originate from different community organization-related models, and thus, this time point should be declared as a change point, or instead 2) all snapshots within the window come from the same model, and thus, there is no change point in that window. As its community organization-related model, GHRG uses generalized

*To whom correspondence should be addressed

hierarchical random graphs. Computational complexity of the method is $O(kn^2 \log n)$, where k is the number of snapshots and n is the number of nodes in snapshots [5].

S2 Methods

S2.1 Our SCOUT approach

Here, we expand our discussion from the main paper on the three main components of SCOUT: objective function (Supplementary Section S2.1.1), consensus clustering (Supplementary Section S2.1.2), and search strategy (Supplementary Section S2.1.3).

S2.1.1 Objective function

For the CSCD problem, in which segmentation parsimony is fixed, an objective function Q should measure partition accuracy of an output A. For the SCD problem, an objective function Q should measure both segmentation parsimony and partition accuracy. We organize the rest of this section as follows. I) We discuss the group of objective functions Q_P that measure only partition accuracy. II) We discuss the group of objective functions Q_B that measure both aspects of the output quality. III) We discuss how to use the above two groups of objective functions to solve the CSCD and SCD problems.

I) To measure only partition accuracy, we define Q_P as the *average snapshot partition quality*:

$$Q_P(A, D) = \frac{1}{k} \sum_{i=0}^{l-1} \sum_{j=t_i}^{t_{i+1}-1} F(p_i, G_j), \tag{1}$$

where F measures the fit of partition p_i to snapshot G_j. Since there is no one universally accepted measure F of how well a given partition p fits a given snapshot $G = (V, E)$, we test four popular such measures F [6]. Let $|p|$ be the number of clusters in partition p. For a given cluster $c \in p$, let n_c be the number of its nodes, let m_c be the number of its internal edges, and let b_c be the number of its boundary edges (edges between the nodes in c and the nodes in $V \setminus c$). We consider the following choices of F: **1)** *Modularity* [7]: $F(p, G) = \frac{1}{2|E|} \sum_{c \in p} (m_c - \mathbb{E}(m_c))$, where $\mathbb{E}(m_c)$ is the expected number of c's internal edges under a configuration model (a random model with the same degree distribution as G). Intuitively, a partition is of high quality with respect to modularity if its clusters are denser than at random. The higher the modularity score, the better the partition accuracy. The remaining three measures are based on the intuition that in a good partition, clusters should have more inside than boundary edges. **2)** *Conductance* [8]: $F(p, G) = \frac{1}{|p|} \sum_{c \in p} \frac{b_c}{2m_c + n_c}$. **3)** *Normalized Cut* [9]: $F(p, G) = \frac{1}{|p|} \sum_{c \in p} (\frac{b_c}{2m_c + n_c} + \frac{b_c}{2(m - m_c) + n_c})$. **4)** *Average-ODF* [10]: $F(p, G) = \frac{1}{|p|} \sum_{c \in p} \frac{1}{|n_c|} \sum_{u \in c} \frac{|\{|(u,v) \in E | v \notin c\}|}{d_u}$, where d_u is the degree of node u. Because for the last three measures, the lower the score, the better the partition accuracy, and because SCOUT aims to *maximize* (rather than minimize) its objective function, SCOUT uses $F'(p, G) = 1 - F(p, G)$ instead of F in its objective function for these three measures.

II) To simultaneously measure both segmentation parsimony and partition accuracy, we define Q_B based on the *model selection problem* [11]. Intuitively, given some $A = (T, P)$ for a dynamic network D, if we use A as a generative model for creating a dynamic network, how well does this model A fit D? On the one hand, the more complex the model (intuitively, the more segments there are in A, i.e., the lower the segmentation parsimony, and also, the more clusters there are in each segment partition), the more likely it is that we will observe a high fit (as measured by the likelihood of D given A, which mostly reflects partition accuracy). On the other hand, the less complex the model, the more likely it is that we will observe a low fit. Given a set of As under consideration (see below), the goal of the model selection problem is to choose $A^\star$ that optimizes some measure of quality over all such As. This measure of quality should balance between the *goodness of the fit* of A to D (mostly partition accuracy) and the *complexity of the model* A (mostly segmentation parsimony).

To solve the model selection problem, we test two popular approaches [11, 12]: **1)** *Akaike Information Criterion* (*AIC*) [13] and **2)** *Bayesian Information Criterion* (*BIC*) [14]. Both approaches compute the goodness of the fit in the same way. They also compute the complexity of the model in the same way.

However, the two approaches differ in how they penalize the objective function by the complexity of the model. We define Q_B using AIC or BIC as follows:

$$Q_B(A, D) = \ell(D|A) - w(D)N_p(A). \tag{2}$$

In the above formula, the goodness of the fit is measured via $\ell(D|A)$, the log-likelihood of D given A (see below). The complexity of the model is measured via $N_p(A)$, the *number of parameters* in A (see below). The above two quantities, $\ell(D|A)$ and $N_p(A)$, are balanced via *penalty weight* $w(D)$. For AIC, $w(D) = 1$. For BIC, $w(D) = \frac{1}{2}\log N_o(D)$, where $N_o(D)$ is the *number of observations* in D (how "large" D is; see below). In general, $w(D)$ is larger in BIC than in AIC, which means that BIC penalizes complex models more heavily than AIC. Intuitively, in our case, this means that BIC prefers outputs with smaller numbers of segments than AIC.

Next, we discuss how to compute $\ell(D|A)$, $N_p(A)$, and $N_o(D)$.

To compute $\boldsymbol{\ell(D|A)}$, we assume that each segment s_i is independent of the others, and thus $\ell(D|A)$ is just the sum of log-likelihoods of the individual segments $\ell(s_i|A)$:

$$\ell(D|A) = \sum_{i=0}^{l-1} \ell(s_i|A), \tag{3}$$

where segmentation $S = \{s_0, s_1, \ldots, s_l\}$ is determined by change point set T of A. To compute $\ell(s_i|A)$, we assume that s_i has an associated *stochastic blockmodel* (see below) and each snapshot G_j within segment s_i is independent given this blockmodel. A stochastic blockmodel is a generative model where probability of an edge is determined by the cluster memberships of its endpoints [15]. The blockmodel contains two parts: a partition p and a *stochastic block matrix* θ of size $|p| \times |p|$, where θ_{c_u,c_v} is the probability of an edge between two nodes u, v from clusters $c_u, c_v \in p$, respectively. The blockmodel associated with s_i is based on the corresponding segment partition p_i and has the stochastic block matrix $\theta^{(i)}$ (see below). Thus, $\ell(s_i|A)$ is just the sum of log-likelihoods of the individual snapshots $\ell(G_j|\hat{\theta}^{(i)}, A)$:

$$\ell(s_i|A) = \sum_{j=t_i}^{t_{i+1}-1} \ell(G_j|\hat{\theta}^{(i)}, p_i), \tag{4}$$

where $\hat{\theta}^{(i)}$ is the maximum likelihood estimator of $\theta^{(i)}_{c_u,c_v}$. That is, $\hat{\theta}^{(i)}_{c_u,c_v}$ is computed as the fraction of the actual and the maximum possible numbers of edges between nodes in cluster c_u and nodes in cluster c_v across all snapshots G_j of segment s_i:

$$\hat{\theta}^{(i)}_{c_u,c_v} = \frac{\sum_{j=t_i}^{t_{i+1}-1} m^{(j)}_{c_u c_v}}{\sum_{j=t_i}^{t_{i+1}-1} n^{(j)}_{c_u c_v}}. \tag{5}$$

In the above formula, $m^{(j)}_{c_u c_v}$ is the number of edges in G_j between nodes in cluster c_u and nodes in cluster c_v, and $n^{(j)}_{c_u c_v}$ is the maximum possible number of such edges. If $c_u \neq c_v$, then $n^{(j)}_{c_u c_v} = n^{(j)}_{c_u} n^{(j)}_{c_v}$, where $n^{(j)}_{c_u}$ and $n^{(j)}_{c_v}$ are the numbers of nodes from G_j that are in clusters c_u and c_v, respectively. If $c_u = c_v$, then $n^{(j)}_{c_u c_v} = \binom{n^{(j)}_{c_u}}{2}$. To compute $\ell(G_j|\hat{\theta}^{(i)}, p_i)$, the log-likelihood of $G_j = (V_j, E_j)$ given $\hat{\theta}^{(i)}$ and p_i, because we are using a stochastic blockmodel, we assume that an edge between each pair of nodes $u, v \in V_j$ is independent of others and its probability is based on the cluster memberships $c_u, c_v \in p_i$ of u, v, respectively. Thus, $\ell(G_j|\hat{\theta}^{(i)}, p_i)$ is just the sum of log-likelihoods of individual edges and non-edges observed in G_j:

$$\ell(G_j|\hat{\theta}^{(i)}, p_i) = \sum_{(u,v)\in E_j} \log \hat{\theta}^{(i)}_{c_u,c_v} + \sum_{(u,v)\notin E_j} \log(1 - \hat{\theta}^{(i)}_{c_u,c_v}). \tag{6}$$

To compute $\boldsymbol{N_p(A)}$, we count the number of values in $\hat{\theta}^{(i)}$s across all segments $s_i, i \in [0, l-1]$. For a given segment s_i, we have one value in $\hat{\theta}^{(i)}$ for each pair of clusters in p_i (including a cluster with itself), so, in total:

$$N_p(A) = \sum_{i=0}^{l-1} (\binom{|p_i|}{2} + |p_i|) = \sum_{i=0}^{l-1} \frac{|p_i|(|p_i|+1)}{2}. \tag{7}$$

To compute $N_o(D)$, we count the number of node pairs in all snapshots $G_j = (V_j, E_j)$ in D (Supplementary Equation 6):

$$N_o(D) = \sum_{j=0}^{k-1} \binom{|V_j|}{2} = \sum_{j=0}^{k-1} \frac{|V_j|(|V_j|-1)}{2}. \tag{8}$$

By combining Supplementary Equations 3, 7, and, for BIC, 8, we can compute $Q_B(A, D)$ in Supplementary Equation 2.

III) Given some consensus clustering method and search strategy (see below), and given the above two groups of objective functions, Q_P and Q_B, we now discuss how to solve the CSCD and SCD problems.

To solve the CSCD problem, we pick as $A^\star$ a solution with the desired number of segments l that maximizes $Q \in \{Q_P, Q_B\}$:

$$A^\star = \underset{|T|=l-1, A\in R}{\text{argmax}} \; Q(A, D), \tag{9}$$

where T is the change point set of A (recall that we need $l-1$ change points to produce l segments) and R is the set of the considered outputs (note that this set is determined by the search strategy; see below). Here, Q can measure either only partition accuracy (i.e., Q_P) or both aspects of the SCD problem (i.e., Q_B).

To solve the SCD problem, we first solve the CSCD problem $\forall l \in [1, k]$ using Q_P or Q_B as described above, and then we pick as $A^\star$ one of these k solutions that maximizes Q_B. Let $R^\star = \{A^\star_{(i)} | i \in [1, k]\}$, where $A^\star_{(i)}$ is the solution of the CSCD problem with i segments (Equation 9). Given $R^\star$, we select $A^\star$ as follows:

$$A^\star = \underset{A\in R^\star}{\text{argmax}} \; Q_B(A, D). \tag{10}$$

Note that if we use the same Q_B when constructing $R^\star$ (Supplementary Equation 9) and when selecting $A^\star$ from $R^\star$ (Supplementary Equation 10), the described procedure for solving the SCD problem is equivalent to directly aiming to find $A^\star$ with the optimal value of Q_B.

S2.1.2 Consensus clustering

Given change point set T, we obtain the set of segment partitions P by applying consensus clustering to each segment. That is, for each segment s_i, we aim to find a single partition p_i that works well for all snapshots in s_i. Note that if s_i contains only one snapshot, consensus clustering is equivalent to simple static network clustering, since there are no multiple snapshots to compute consensus for; yet, for consistency, we still refer to such clustering process as consensus clustering. Intuitively, the chosen consensus clustering method should align with the objective function, meaning that, for a given change point set T, consensus clustering should aim to find the set P of segment partitions that maximize the objective function Q. We consider three consensus clustering methods: *sum graph* [4], *Average-Louvain* [4], and *consensus matrix* [16].

Sum graph. An intuitive way to perform consensus clustering for a given segment s_i is to first construct a special graph that "summarizes" the topology of all snapshots in s_i and then find community organization in this "summary" graph under the hypothesis that this organization will fit well all snapshots in s_i. Here, we construct this "summary" graph for s_i simply as a *sum graph*, a weighted graph whose adjacency matrix is the sum of the adjacency matrices of all snapshots in s_i [4]. Then, we use a static community detection method that can handle weighted graphs to find a partition in this sum graph. We test seven popular static community detection methods [17]: **1)** *Fast Modularity* [18]: the method starts with each node as a singleton community, and then at every iteration it merges two communities to greadily optimize modularity. **2)** *Label Propagation* [19]: the method starts with each node as a singleton community (referred to as a *label*), and then at every iteration each node adopts the label used by the majority of its neighbors. **3)** *Leading Eigenvector* [20]: the method optimizes modularity based on the eigenspectrum of a modularity matrix (a matrix analogous to graph Laplacian in graph partitioning). **4)** *Infomap* [21]: the method aims to find a partition minimizing the expected description length of a random walker trajectory. **5)** *Walktrap* [22]: the method finds a partition based on the intuition that short random walks tend to get "trapped" in the same community, since, intuitively, there are many edges pointing inside the community and only few pointing outside. **6)** *Louvain* [23]: the method starts with each node as a singleton community and then repeatedly performs two phases: greedily optimizing modularity by moving nodes to neighboring communities and

constructing a new graph with communities as nodes. **7)** *Stabilized Louvain* [24]: a modification of Louvain algorithm for snapshot clustering that aims to produce stable partitions (i.e., prevent two snapshots with similar topologies from having dissimilar partitions); to achieve stability, the method clusters a snapshot at time t via Louvain algorithm initialized with the partition obtained for the snapshot at time $t-1$.

Average-Louvain. This method aims to find a segment partition p_i that maximizes average modularity over all snapshots in s_i [4]. To achieve this, the method uses a modification of Louvain algorithm for static community detection (see above). Recall that Louvain method contains two phases. In Average-Louvain, the first phase is modified so that the modularity gain of each move is computed as the average gain of this move across all snapshots in the given segment. The second phase, constructing a network of communities, is modified so that the same transformation is performed independently on all snapshots within the given segment. Thus, all snapshots have the same partition, which becomes p_i.

Consensus matrix. This method aims to find a segment partition p_i directly from the partitions of snapshots in s_i [16]. That is, given individual snapshot partitions as input, the method computes a *consensus matrix* M based on the co-occurrence of nodes in clusters of the input partitions. Specifically, entry M_{ij} of this matrix indicates the fraction of the input partitions in which nodes i and j are in the same cluster. Matrix M, which can be thought of as a weighted graph, can then be clustered by some static community detection method to produce a consensus partition. To compute snapshot partitions as well as to cluster M, we use the same static community detection methods as for the sum graph approach above.

S2.1.3 Search strategy

We test three strategies for exploring the space of possible change point sets: the *exhaustive search*, *top-down search*, and *bottom-up search*. Each strategy first produces one best solution for each possible number of segments for the CSCD problem (Supplementary Equation 9), which are then used to solve the SCD problem (Supplementary Equation 10). The first strategy is aimed at producing a globally optimal solution at the expense of larger running time, while the last two are heuristics aimed at producing a good solution in a faster manner. Below, for each strategy, we discuss how the strategy works and its "conceptual" computational complexity. By "conceptual", we mean that we express the running time of a given strategy in terms of the number of times that consensus clustering is performed. We do this because: 1) performing consensus clustering is SCOUT's most computationally intensive step whose running time dominates all other steps, and 2) we vary consensus clustering methods within SCOUT, and thus, we account only for the number of times that consensus clustering is performed, since the actual computational complexity of performing each consensus clustering depends on the chosen clustering method.

Exhaustive search. This strategy aims to find a globally optimal solution under the chosen consensus clustering method by exhaustively searching through the space of all possible Ts. There are $\binom{k-1}{l-1}$ ways to group all k snapshots of D into $l \in [1,k]$ segments. Thus, for all ls, the exhaustive search needs to explore the total of $\sum_{i=1}^{k}\binom{k-1}{i-1} = 2^{k-1}$ different segmentations (or, equivalently, change point sets).

To reduce the computational complexity, we use dynamic programming, as follows. The search contains k iterations. Consider the ith iteration ($i \in [1,k]$). Let $D_{[q,r]} = \{G_q, G_{q+1}, \ldots, G_{r-1}, G_r\}$ be all consecutive snapshots of D from time q to time r, inclusively. The goal of the ith iteration is to solve the SCD problem for $D_{[0,i-1]}$ (i.e., for the first i snapshots of D). For $i = k$, this means obtaining the solution for the whole network $D = D_{[0,k-1]}$. Recall from Supplementary Section S2.1.1 that in order to solve the SCD problem, we first need to solve the CSCD problem for each possible number of segments (Supplementary Equation 10). That is, in the ith iteration, $\forall l \in [1,i]$, we need to find the optimal solution $A^{\star}_{i,l}$ for $D_{[0,i-1]}$ that has l segments. Next, we discuss how to find such $A^{\star}_{i,l}$. Any solution A for $D_{[0,i-1]}$ that has l segments can be split into two parts with respect to start time t of its last segment: **1)** the part with the first $l-1$ segments, which can be thought of as a solution for prefix $D_{[0,t-1]}$ of $D_{[0,i-1]}$ and **2)** the part with the last l^{th} segment, which can be thought of as a solution for suffix $D_{[t,i-1]}$ of $D_{[0,i-1]}$. Conversely, solution A can be constructed by combining the above two parts. Now, since $A^{\star}_{i,l}$ is the optimal solution (with respect to the given consensus clustering method), its corresponding two parts should be optimal too. That is, the first part should be the optimal solution with $l-1$ segments for $D_{[0,t-1]}$ (which is exactly $A^{\star}_{t,l-1}$, and which is known from the earlier $t^{(th)}$ iteration), and the second part should be the optimal solution with one segment for $D_{[t,i-1]}$ (which is a solution with just one segment, whose only segment partition can be obtained by

performing consensus clustering of $D_{[t,i-1]}$). So, we know the first part of $A^{\star}_{i,l}$ from one of the previous iterations and can compute its second part in the current iteration. However, in order to actually construct $A^{\star}_{i,l}$ from the above two parts, we need to know start time t of its last segment. If $l = 1$, there is only one value for t (namely, $t = 0$), since the lth (i.e., the only) segment should encompass the whole $D_{[0,i-1]}$. If $l \in [2, i]$, t can take any value from $l - 1$ (in which case the $l - 1$ segments in the first part are all singletons) to $i - 1$ (in which case the lth segment in the second part is a singleton). So, for $l \in [2, i]$, to find t, we simply test all of its possible values and pick the one that produces the solution that maximizes the objective function (Supplementary Figure S1).

Given the above procedure, we next discuss its computational complexity. Consider the ith iteration ($i \in [1, k]$). In this iteration, we need to find $A^{\star}_{i,l}$ for each $l \in [1, i]$. Recall from the above paragraph that each $A^{\star}_{i,l}$ is constructed from two parts, and only for the second part, corresponding to its last segment and starting at some time point t, we do not know the corresponding segment partition and thus need to obtain this partition in the current iteration. For $l = 1$, we test only one value of t ($t = 0$), and for $l \in [2, i]$, we test all values of t from $l - 1$ to $i - 1$. For each t, we need to obtain consensus partition for suffix $D_{[t,i-1]}$ of $D_{[0,i-1]}$. Clearly, different values of l can deal with the same t, i.e., the same suffix, and for a given suffix we need to obtain its consensus partition only once. Overall, there are i distinct values of t, from 0 to $i - 1$, and thus i distinct suffixes of $D_{[0,i-1]}$ for which we need to obtain consensus partitions. Thus, in the ith iteration, we need to perform consensus clustering for the total of i times, once for each such suffix. Therefore, for all k iterations, we perform consensus clustering $\sum_{i=1}^{k} i = \frac{k^2+k}{2} = O(k^2)$ times.

Top-down search. This strategy aims to find a good solution by greedily searching through the set of possible Ts in a top-down manner. The search contains k iterations. We start with one segment of length k and at each subsequent iteration split one of the existing segments into two parts in a locally optimal way with respect to the chosen objective function, until we reach k singleton segments (Supplementary Figure S2). Since the search starts with one segment and since in each iteration the number of segments is increased by one, the solution obtained in the ith iteration is the solution for the CSCD problem with i segments. Hence, after k iterations, we have one solution for the CSCD problem for each possible number of segments, which can be used to solve the SCD problem (Supplementary Section S2.1.1). More specifically, the top-down search works as follows. In the first iteration, we have only one segment, and we perform consensus clustering once for this segment. Then, at the start of the ith iteration ($i \in [2, k]$), we have $i - 1$ segments from the previous iteration (denoted as $\{s_0^{(i-1)}, s_1^{(i-1)}, \ldots, s_{i-2}^{(i-1)}\}$), and we aim to split one of these $i - 1$ segments into two parts by inserting a new change point $t^{\star}_{(i)}$, in order to produce i segments. There are $k - i + 1$ candidate time points t' for $t^{\star}_{(i)}$: the total of k time points minus $t_0 = 0$ and minus $i - 2$ change points selected in the previous iterations. Out of these candidates, we choose the one that maximizes gain (or minimizes loss) in our objective function.

To reduce the computational complexity, we show that we can reuse in each iteration the results from the previous iterations. In the ith iteration ($i \in [2, k]$), each candidate time point t' leads to splitting some current segment $s_j^{(i-1)}$ into two parts. So, for a given t, we need to know two segment partitions: one for the first half of segment $s_j^{(i-1)}$ (that ends at time $t-1$) and one for the second half of segment $s_j^{(i-1)}$ (that starts at time t). We do not necessarily need to compute these segment partitions in the current iteration, since we can reuse the results from the previous iterations, as follows. Consider the next $(i+1)^{st}$ iteration. Let $s_{r_i}^{(i)}$ be the segment that was split in the ith iteration (by inserting $t^{\star}_{(i)}$). In the $(i+1)^{st}$ iteration, all segments except the two resulted from splitting $s_{r_i}^{(i-1)}$ are the same as in the ith iteration (Supplementary Figure S2). Thus, when testing candidate time points inside these unchanged segments, we can just reuse segment partitions from the previous iterations. So, in the $(i + 1)^{st}$ iteration, we only need to perform consensus clusterings for those candidate time points that are within the two newly created segments (Supplementary Figure S2). Such time points are all time points inside the segment that was split in the ith iteration except its start point and except already taken $t^{\star}_{(i)}$.

The above described reuse of previous consensus clusterings generally allows for reducing the complexity compared to the exhaustive search. However, in the worst case, the complexity of the top-down search is still $O(k^2)$: if each new change point $t^{\star}_{(i)}$ is selected as the earliest one (i.e., if $t^{\star}_{(i)} = i - 1$), the overall number of performed consensus clusterings is $1 + 2(k - 1) + \sum_{i=3}^{k}(k - i + 1) = \sum_{i=1}^{k}(k - i + 1) = (k^2 + k)/2 = O(k^2)$. Nevertheless, even though this is the same theoretic complexity as for the exhaustive search above, in practice,

the top-down search is faster (Supplementary Section S3).

Bottom-up search. This strategy aims to find a good solution by greedily searching through the set of possible Ts in a bottom-up manner. The search contains k iterations. We start with k singleton segments and at each subsequent iteration merge two existing adjacent segments in a locally optimal way (with respect to the chosen objective function), until we reach one large segment of length k. Since the search starts with k segments and since in each iteration the number of segments is decreased by one, the solution obtained in the ith iteration is the solution for the CSCD problem with $k-i+1$ segments. Hence, after k iterations, we have one solution for the CSCD problem for each possible number of segments, which can be used to solve the SCD problem (Supplementary Section S2.1.1). More specifically, the bottom-up search works as follows. In the first iteration, we perform consensus clustering k times, once for each segment. Then, at the start of the ith iteration ($i \in [2,k]$), we have $k-i+2$ segments from the previous iteration (denoted as $\{s_0^{(i-1)}, s_1^{(i-1)}, \ldots, s_{k-i+1}^{(i-1)}\}$), and we aim to merge some two adjacent segments $(s_{r_i}^{(i)}, s_{r_i+1}^{(i)})$ from these $k-i+2$ segments in order to produce $k-i+1$ segments. There are $k-i+1$ candidate segment pairs $(s_j^{(i)}, s_{j+1}^{(i)})$ (or, equivalently, $k-i+1$ candidate change points to be removed), since there are $k-i+2$ segments and since we consider only adjacent segment pairs. Out of these candidates, we choose the one that maximizes gain (or minimizes loss) in our objective function.

To reduce the computational complexity, we next show that we can reuse in each iteration the results from the previous iterations. In the ith iteration ($i \in [2,k]$), each candidate segment pair $(s_j^{(i)}, s_{j+1}^{(i)})$ leads to merging segments $s_j^{(i)}$ and $s_{j+1}^{(i)}$. So, for a given segment pair $(s_j^{(i)}, s_{j+1}^{(i)})$, we need to know one segment partition for the merged segment $s_q^{(i)} \cup s_{q+1}^{(i)}$. We do not necessarily need to compute these segment partitions in the current iteration, since we can reuse the results from the previous iterations, as follows. Consider the next $(i+1)^{st}$ iteration. In the $(i+1)^{st}$ iteration, all segments except the newly created one (i.e., the segment resulting from merging the two segments chosen in the ith iteration) are the same as in the ith iteration (Supplementary Figure S3). Thus, when testing candidate segment pairs not involving the new segment, we can just reuse segment partitions from the previous iteration. So, in the $(i+1)^{st}$ iteration, we only need to perform consensus clusterings for those candidate segment pairs that involve the new segment. There are at most two such segment pairs, since we consider only adjacent segments.

In the first iteration, we perform consensus clustering k times (once for each snapshot). In the second iteration, we perform consensus clustering $k-1$ times (once for each pair of adjacent snapshots). For all subsequent iterations, as discussed above, we perform consensus clustering at most twice. Therefore, for all k iterations, we need to perform consensus clustering at most $k + (k-1) + \sum_{i=3}^{k} 2 = 4k-5 = O(k)$ times.

S2.2 Experimental setup

S2.2.1 Methods for comparison

Here, we expand our discussion from the main paper on the methods that we use in our experiments and their parameters.

GraphScope does not accept any user-defined parameters. Note that GraphScope was originally designed to work only with bipartite graphs, and thus it produces two separate partitions. Hence, in order to handle unipartite graphs such as the data from our study, we constrain GraphScope to produce only one partition [1]. The method can solve only the SCD problem. We use a publicly available implementation of GraphScope [25].

Multi-Step performs an agglomerative clustering of the snapshots, merging them into segments to produce a hierarchical tree. To get a solution for the CSCD problem, we cut the tree at the level that results in the desired number of segments. To get a solution for the SCD problem, we first test Multi-Step's suggested procedure of cutting the tree at the level above which the highest segment similarity is negative. We note that this procedure is used as a way to stop the merging process early in order to prevent meaningless merges, rather than as a way to select the best segmentation [4]. Importantly, as we show, this procedure consistently underestimates the number of segments that actually exist in the data. To address this, we introduce a user-specified segment similarity threshold θ, and instead of stopping the merging process as soon as the highest similarity becomes negative, we instead stop this process as soon as the highest similarity becomes less than θ. That is, in Multi-Step's default procedure, $\theta = 0$. To give Multi-Step the best-case

advantage, we vary θ from 0 to 2 in increments of 0.1 (note that since the similarity between two segments is computed as the sum of modularities of the two segments, and since the maximum value of modularity for any segment is 1, the maximum possible segment similarity value is 2; Supplementary Section S1). However, we find that the optimal threshold θ is network-specific, and a threshold that works well for one network may not work well for other networks. Note that by trying different values of θ, essentially, we allow Multi-Step to try solutions with different numbers of segments. Thus, when the ground truth number of segments is known, we simply provide this information as input to Multi-Step. That is, instead of using Multi-Step to solve the SCD problem, we use it to solve the CSCD problem where we set l to match the ground truth number of segments. This is not fair to the other methods (including SCOUT), which aim to solve the full SCD problem (and thus automatically find l that ideally matches the ground truth value). Yet, this is what we have to do in order to include Multi-Step into the comparison, since this method can reliably solve only the CSCD problem. We use a publicly available implementation of Multi-Step [4].

GHRG relies on a sliding window approach with the length of the window w being a user-defined parameter. We test $w \in \{4, 8, 12\}$. For each segment, GHRG results in a generalized hierarchical tree model instead of a partition. To obtain a partition from this model, we cut the tree in a way that maximizes modularity [23]. The method can solve only the SCD problem. We use a publicly available implementation of GHRG [5].

SCOUT contains three main components: objective function, consensus clustering, and search strategy. We test different choices for these components: two objective functions (with four choices for Q_P and two choices for Q_B; Supplementary Section S2.1.1), three consensus clustering approaches (with seven choices for each of sum graph and consensus matrix and one choice for Average-Louvain; Supplementary Section S2.1.2), and three search strategies (Supplementary Section S2.1.3). To allow for experimenting with the different parameter choices, our initial SCOUT implementation focuses on flexibility (to allow for easily testing various parameter choices for the method's components) rather than on running time. However, once we finalize the most optimal (i.e., accurate yet efficient) choice of the parameters (Supplementary Section S3), we develop a faster parallel SCOUT implementation tailored for the selected parameters and aimed at reducing the running time. So, when comparing SCOUT against the other approaches, we use its latter fast implementation. As discussed in Supplementary Section S2.1.1, SCOUT can solve both the CSCD and SCD problem.

S2.2.2 Datasets

Here, we expand our discussion from the main paper on synthetic and real-world networks that we use for our experiments.

Synthetic networks. Here, we describe in detail our synthetic network generation process. We assume that the following are provided as input by the user: the number of snapshots k, the number of segments l, the number of nodes in each snapshot n, the minimum required number of nodes in each cluster c_{min}, and two parameters c_{in} and c_{out} that control intra- and inter-community edge density of the snapshots. In our experiments, we set $c_{min} = 5$ when $n = 50$, $c_{min} = 10$ when $n = 100$ and $c_{min} = 50$ when $n \in \{500, 1000\}$. Also, we set $c_{in} = 32$ and $c_{out} = 8$. The process of generating a synthetic dynamic network with these parameters contains four steps (Supplementary Figure S4). In the first three steps, we generate the ground truth $A^{(gt)} = (T^{(gt)}, P^{(gt)})$, and in the last step, we use $A^{(gt)}$ to actually generate snapshots of D. Intuitively, we: **1)** generate the set of change points $T^{(gt)}$ to define segments, **2)** create a special auxiliary graph describing how segment partitions evolve from segment to segment, **3)** use this graph to generate the actual segment partitions $P^{(gt)}$, and **4)** use a stochastic blockmodel to generate snapshots of D, based on the idea that snapshots within the same segment (as defined by $T^{(gt)}$) have the same community organization (as defined by the corresponding segment partition from $P^{(gt)}$). The details of the four steps are as follows:

1) To create a segmentation with l segments, we randomly sample $l-1$ change points $T^{(gt)}$ from $[1, k-1]$. For example, in Supplementary Figure S4, we select two change points $t_1^{(gt)}$ and $t_2^{(gt)}$ to create three segments. Thus, at the end of this step, we have the change point set $T^{(gt)}$.

2) In this and the next step, we aim to generate segment partitions $P^{(gt)}$, as follows. To achieve this, we use a special *partition graph* G_P. In this step, we intuitively define G_P and describe how we generate G_P and in the next step we describe how use G_P to create $P^{(gt)}$.

We need to create $G_P = (V_P, E_P)$ as a weighted directed l-partite graph (i.e., $V_P = V_P^{(0)} \cup V_P^{(1)} \cup \cdots \cup V_P^{(l-1)}$) with every edge in E_P having form (u, v), where $u \in V_P^{(i-1)}$ and $v \in V_P^{(i)}$ for some $i \in [1, l-1]$. To

avoid confusion between nodes of G_P and nodes of D, we refer to nodes of G_P as *supernodes*. Intuitively, once we generate G_P at the end of this step, each of its supernode sets $V_P^{(i)}$ will correspond to segment partition $p_i^{(gt)}$ of $A^{(gt)}$, with each supernode of $V_P^{(i)}$ corresponding to some cluster of $p_i^{(gt)}$. For example, in Supplementary Figure S4, $V_P^{(0)}$ has four supernodes, so $p_0^{(gt)}$ will have four clusters. Each edge between two supernodes of G_P intuitively means that the two clusters corresponding to these two supernodes have shared members (see the next step for more details).

We construct G_P starting with empty sets V_P and E_P. To create V_P, we add a random number $r_i \in [2, \lfloor n/c_{min} \rfloor]$ of supernodes to each $V_P^{(i)} \subset V_P$, $i \in [0, l-1]$. Recall from above that c_{min} is the user-defined minimum required number of nodes in a cluster. Thus, the limits for r_i mean that we want segment partition corresponding to $V_P^{(i)}$ to have at least two clusters, while at the same time we want to prevent it from having too many clusters. To create E_P, we randomly create edges between each pair of adjacent sets $V_P^{(i-1)}$ and $V_P^{(i)}$ ($i \in [1, l-1]$) as follows. For each edge, we randomly pick its two endpoints: one supernode in $V_P^{(i-1)}$ and one supernode in $V_P^{(i)}$. When adding edges to E_P, we aim to satisfy the following three conditions: **a)** the edges do not form a perfect matching between $V_P^{(i-1)}$ and $V_P^{(i)}$ (i.e., segment partitions $p_{i-1}^{(gt)}$ and $p_i^{(gt)}$ are not identical), **b)** each supernode in $V_P^{(i-1)}$ has at least one outgoing edge and each supernode in $V_P^{(i)}$ has at least one incoming edge (i.e., clusters cannot appear/disappear), and **c)** for each edge $e = (u, v)$, assuming d^+ and d^- is the outdegree and indegree of a supernode, respectively, one of the following holds: (i) $d^+(u) = 1$ and $d^-(v) > 1$ (i.e., the cluster corresponding to u merges with at least one other cluster into the cluster corresponding to v; e.g., the top right edge in Supplementary Figure S4), (ii) $d^+(u) > 1$ and $d^-(v) = 1$ (i.e., the cluster corresponding to u is split into several clusters including the cluster corresponding to v; e.g., the bottom right edge in Supplementary Figure S4), or (iii) $d^+(u) = d^-(v) = 1$ (i.e., the cluster corresponding to u and the cluster corresponding to u have the same members; e.g., the top left edge in Supplementary Figure S4). Intuitively, for two adjacent segment partitions, edges satisfying (i) correspond to merges of clusters, edges satisfying (ii) correspond to splits of cluster, and edges satisfying (iii) correspond to unchanged clusters. Note that in general, the condition (iii) can be relaxed; we include it to provide further coherence between individual clusters so that their changes can be described via three simple events (merge, split, and continuation).

3) Using G_P from the previous step, we now generate the set of segment partitions $P^{(gt)}$, as shown in Supplementary Algorithm S2. Intuitively, each edge in G_P means that some two clusters share members. We construct segment partitions one by one, starting from the partition of the first segment. For each segment partition, we determine the membership of a given cluster based on the incoming edges of the corresponding supernode and the previous segments partition. At the end of this step, we have the complete $A^{(gt)} = (T^{(gt)}, P^{(gt)})$.

4) We use $A^{(gt)} = (T^{(gt)}, P^{(gt)})$ to generate snapshots of D as follows. Intuitively, we aim to generate each snapshot based solely on the corresponding segment partition. Thus, to generate a snapshot G_j of a given segment s_i, we use the stochastic blockmodel with partition $p_i^{(gt)}$ and stochastic block matrix θ (Supplementary Section S2.1.1). We use the same θ for all segments, with $\theta_{c_u c_v} = c_{in}/n$ if $c_u \neq c_v$ and $\theta_{c_u c_v} = c_{in}/n$ otherwise. So, to generate a snapshot G_j of segment s_i, for each pair of nodes in G_j, we independently place an edge between the two nodes with probability c_{in}/n if they are in the same cluster in p_i and with probability c_{out}/n otherwise [26]. Note that even though all snapshots in segment s_i are created based on the same segment partition p_i, they still likely differ from each other due to randomness in the stochastic blockmodel. At the end of this step, we have all snapshots G_j of D, and, moreover, the structure of D reflects the ground truth $A^{(gt)} = (T^{(gt)}, P^{(gt)})$.

Real-world networks. We consider six different publicly available real-world dynamic networks. **1)** *Hypertext* [27] network contains information about face-to-face proximity of attendees of the Hypertext 2009 conference. The nodes correspond to people, and there is an edge between two people if they were close to each other within a given time interval, as measured by wearable radio badges. This network has $T^{(gt)}$ that corresponds to the list of events from the conference program [27]. **2)** *AMD Hope* [28] network contains information about co-location of attendees of The Last HOPE conference in 2008. The nodes correspond to people, and there is an edge between two people if they were located in the same room at the same time. This network has $T^{(gt)}$ that corresponds to the featured/keynote talks and social events [28]. **3)** *High School* [29] network contains information about proximity of students in a high school during one work week in

2013. The nodes and edges are added in the same way as in Hypertext network. This network does not have $T^{(gt)}$. **4)** *Reality Mining* [30] network contains information about social interactions of university students and faculty during 2004-2005 academic year. The nodes correspond to people, and there is an edge between two people if there was a phone call between them in a given time interval. This network has $T^{(gt)}$ that corresponds to the list of events from the academic calendar [5]. **5)** *Enron* [31] network contains information about email communication of employees of the Enron corporation during the 2000-2002 period. The nodes correspond to people, and there is an edge between two people if there was an email between them in a given time interval. This network has $T^{(gt)}$ that corresponds to the list of company-related events from the news sources [5]. **6)** *Senate* [32] network contains information about voting similarities of United States senators during the 1789-2015 period (i.e., for 113 Congresses). The nodes correspond to states, and there is an edge between two states if the voting similarity between the corresponding senators in a given time interval is high enough [32]. This network does not have $T^{(gt)}$. This is because for *Senate* network we cannot use the list of historic events as a formal ground truth change point set, since it is not clear how to objectively select a fixed number of them (i.e., how to determine which events are more important than others and how many of the most important events should be considered). For statistics of the real-world networks, see Supplementary Table S2.

S2.2.3 Evaluation measures

Here, we expand our discussion from the main paper on the evaluation measures.
I) We start by discussing the three output similarity measures.
Segmentation similarity Sim_T. To measure Sim_T between $A^\star$ and $A^{(gt)}$, intuitively, we first construct for each of them a special time point partition $\mathcal{P}_T$ that captures how the snapshots of D are grouped into segments. For example, for $A^\star$ and $A^{(gt)}$ in Supplementary Figure S5, $\mathcal{P}_T^\star$ contains three clusters ($\{0,1,2,3\}$, $\{4,5,6\}$, and $\{7,8,9\}$) and $\mathcal{P}_T^{(gt)}$ contains four clusters ($\{0,1\}$, $\{2,3,4,5\}$, $\{6,7\}$, and $\{8,9\}$). Then, we measure similarity between the two resulting time point partitions via an existing partition similarity measure (see below). Formally, for a given $A = (T,P)$, we construct its time point partition $\mathcal{P}_T$ as a partition of the set $[0, k-1]$ into l clusters $c_i, i \in [0, l-1]$, where cluster $c_i = \{t_i, t_i+1, \ldots, t_{i+1}-1\}$. Then, to compute Sim_T between $A^\star$ and $A^{(gt)}$, we measure similarity of their corresponding time point partitions $\mathcal{P}_T^\star$ and $\mathcal{P}_T^{(gt)}$, as follows:

$$Sim_T(A^\star, A^{(gt)}) = H(\mathcal{P}_T^\star, \mathcal{P}_T^{(gt)}), \tag{11}$$

where H can be any partition similarity measure (see below).
Partition similarity Sim_P. To measure Sim_P between $A^\star$ and $A^{(gt)}$, intuitively, we first measure for each snapshot of D similarity between its corresponding segment partitions in $A^\star$ and $A^{(gt)}$ via an existing partition similarity measure (see below). For example, for $A^\star$ and $A^{(gt)}$ in Supplementary Figure S5, for snapshot G_0, we measure similarity between $p_0^\star$ and $p_0^{(gt)}$ (since G_0 belongs to the first segment in $A^\star$ and to the first segment in $A^{(gt)}$), while for snapshot G_2, we measure similarity between $p_0^\star$ and $p_1^{(gt)}$ (since G_2 belongs to the first segment in $A^\star$ and to the second segment in $A^{(gt)}$). Then, we average the results over all snapshots. Formally, to compute Sim_P between $A^\star$ and $A^{(gt)}$, we introduce *average snapshot partition similarity*, as follows:

$$Sim_P(A^\star, A^{(gt)}) = \frac{1}{k}\sum_{j=0}^{k-1} H(p^\star_{seg(j,T^\star)}, p^{(gt)}_{seg(j,T^{(gt)})}), \tag{12}$$

where H can be any partition similarity measure (see below) and $seg(j,T)$ is the function that returns the index of the segment containing snapshot G_j under the segmentation induced by change point set T (i.e., $seg(j,T) = i \iff G_j \in s_i$). Note that even though Sim_P focuses on the partition aspect of the SCD problem, it still implicitly relies on the segmentation aspect via the above *seg* function.
Overall similarity Sim_B. To measure Sim_B between $A^\star$ and $A^{(gt)}$, intuitively, we first construct for each of them a special node-time partition $\mathcal{P}_B$ that simultaneously captures how snapshots are grouped by Ts and how nodes are grouped by Ps. For illustrations of node-time partitions of $A^\star$ and $A^{(gt)}$, see Supplementary Figure S5. Then, we measure similarity between the two resulting node-time partitions via an existing partition similarity measure (see below). Formally, for a given $A = (T,P)$, we construct a node-time partition $\mathcal{P}_B$ as a partition of the set $\{(u,t) | u \in V_t, t \in [0, k-1]\}$ into $\sum_{i=0}^{l-1} |p_i|$ clusters. Two

node-time pairs (u_1, t_1) and (u_2, t_2) are clustered together in $\mathcal{P}_B$ if their time points t_1 and t_2 belong to the same segment (i.e., if t_1 and t_2 are in the same cluster in $\mathcal{P}_T$, or $seg(t_1, T) = seg(t_2, T)$) and if their nodes u_1 and u_2 belong to the same cluster in the corresponding segment partition (i.e., if u_1 and u_2 are in the same cluster in $p_{seg(t_1,T)}$). Then, to compute Sim_B between $A^\star$ and $A^{(gt)}$, we measure similarity between their corresponding node-time partitions $\mathcal{P}_B^\star$ and $\mathcal{P}_B^{(gt)}$, as follows:

$$Sim_B(A^\star, A^{(gt)}) = H(\mathcal{P}_B^\star, \mathcal{P}_B^{(gt)}), \tag{13}$$

where H can be any partition similarity measure.

Next, we describe the four partition similarity measures H that we use: **1)** *Normalized Mutual Information* (*NMI*) [33] – a measure of similarity based on the mutual information (MI), normalized to have values in $[0, 1]$. **2)** *Adjusted Mutual Information* (*AMI*) [33] – an adjusted for chance version of MI. **3)** *Adjusted Rand Index* (*ARI*) [33] – an adjusted for chance version of the Rand Index, a measure of similarity based on counting pairs of observations assigned to the same cluster or different clusters in two partitions. For AMI and ARI, the adjustment for chance means correction for chance agreement between two partitions [33]. **4)** *V-Measure* (*VM*) [34] – the harmonic mean of *homogeneity* (whether the first partition groups together *only* those objects that are grouped together in the second partition) and *completeness* (whether the first partition groups together *all* those objects that are grouped together in the second partition). Note that for all of the above measures, a higher value means higher similarity, with two identical partitions having similarity of one.

II) Next, we describe the three measures of change point classification accuracy that we use: **1)** $\forall i \in [1, k]$, *precision* is the fraction of the top ranked i time points that are ground truth change points (i.e., that belong to $T^{(gt)}$), and *recall* is the fraction of all ground truth change points that are among the top ranked i time points. To summarize the values of precision and recall over all possible values of i, we compute the *area under the precision-recall curve* (*AUPR*). **2)** $\forall i \in [1, k]$, *F-score* is the harmonic mean of precision and recall (that is, F-score balances the two quantities). We report the *maximum F-score* over all values of i. **3)** $\forall i \in [1, k]$, *sensitivity* is equal to recall and *specificity* is the fraction of ground truth non-change points (corresponding to the complement of $T^{(gt)}$) that are not among the top ranked i time points. To summarize the values of sensitivity and specificity over all possible values of i, we compute the *area under the receiver operator characteristic curve* (*AUROC*).

We obtain the ranked list of all time points for each of the considered methods as follows. For a given method, to get its ranked list, we compute the score (see below) for each time point $t \in [1, k-1]$, such that the time points with lower scores are ranked higher (i.e., are more "change point-like"). Note that here we exclude from consideration time point $t_0 = 0$, because, by definition, for any method, t_0 always denotes the start of the first segment, and hence including it into comparison does not provide any method-specific information. If a method is capable of solving the CSCD problem, we compute the scores of the time points using the solutions with all possible numbers of segments for the CSCD problem. Specifically, let $A^\star_{(i)} = (T^\star_{(i)}, P^\star_{(i)})$ be the solution with i segments for the CSCD problem. Intuitively, if a given time point t is a change point in $A^\star_{(i)}$ (i.e., if $t \in T^\star_{(i)}$), this means that the method, when asked to select only $i-1$ change points (i.e., to produce i segments), chooses t as one of these $i-1$ change points. Hence, to capture the intuition that more "change point-like" time points appear in the solutions with smaller number of segments, we compute the score of a given time point t as the smallest number of segments for which t appears as a change point in the corresponding CSCD solution (i.e., $score(t) = \min\{i | i \in [1, k], t \in T^\star_{(i)}\}$). So, the highest ranked time point will appear in the solution with two segments (for which only one time point is selected as change point), while the lowest ranked time point will appear only in the solution with k segments (for which all time points are selected as change point). Since among the considered methods only Multi-Step and SCOUT can solve the CSCD problem (Supplementary Section S2.2.1), the above procedure can only be used for these two methods. For the remaining two methods, GraphScope and GHRG, we use alternative strategies for extracting their ranked lists, as follows. GraphScope, at each step, marks a time point t as a change point if the community organization of G_t does not match well the community organization of the current segment (Supplementary Section S1). The match is measured via the MDL principle, as the difference of the cost of encoding the current segment and G_t together and the cost of encoding them separately. Intuitively, the smaller the difference, the "cheaper" it is to add G_t to the current segment, and when the difference is negative, G_t is added to the current segment. Thus, we use the difference of the encoding costs as the score

of t. GHRG performs a statistical test at each step to determine whether the current window contains a change point (Supplementary Section S1). A change point is detected if its corresponding p-value is smaller than the chosen threshold. Intuitively, the smaller the p-value, the more confident the method is that the given time point t is a change point. Thus, we use the p-value as the score of t.

S2.2.4 Statistical significance of two methods' performance difference

Given a synthetic network configuration, evaluation measure, and a pair of methods, we compute the statistical significance of the difference between the performance of the two methods as follows. For each method, we create a list containing performance scores of the method for all network instances of the synthetic network configuration. Since we know which score in a given list corresponds to which network instance, we use *paired* t-test to compute the statistical significance of the difference between the two lists produced by the two methods. There are four possible outcomes: **1)** method 1 outperforms method 2, and the improvement is statistically significant, **2)** method 1 outperforms method 2, but the improvement is not statistically significant, **3)** method 2 outperforms method 1, but the improvement is not statistically significant, and **4)** method 2 outperforms method 1, and the improvement is statistically significant (note that outcomes 2 and 3 also cover the case when the two methods' scores are tied). By statistically significant, we mean that the p-value that results from paired t-test is below a threshold. We test three p-value thresholds: 0.05, 0.01, and 0.001.

S2.2.5 Statistical significance of SCOUT's top ranked change points in the context of the case study on human aging

We measure probability of obtaining by chance the SCOUT's result from Figure 5 in the main paper as follows. SCOUT identifies one change point in each of the following three age intervals: 33-37, 48-52, and 60-80. Given the available time points in the entire data, there are four ways to select a time point within the first age interval (i.e., 33, 34, 36, and 37), two ways to select a time point within the second age interval (i.e., 48 and 52), and seven ways to select a time point within the third age interval (i.e., 64, 66, 69, 70, 74, 75, and 80). Since intervals 33-37, 48-52, and 60-80 are non-overlapping, a single time point can belong to only one interval at a time. Hence, by the product rule of counting, there are $4 \times 2 \times 7 = 56$ ways to select three time points so that each of the three points falls within one of the three intervals while each interval is covered by one of the points. Since there are 23 available time points in the entire data, there are $\binom{3}{23} = 1771$ ways to select three of them. So, the probability of obtaining SCOUT's result by chance is $56/1771 = 0.03162$.

S3 Results: the effect of method parameter choices

We perform all experiments from this section on synthetic networks, since they have the known ground truth knowledge embedded into them (Supplementary Section S2.2.2). In particular, due to high computational complexity of some of the existing methods and a large number of performed tests, in this section, we use the smallest synthetic data with 50 nodes per snapshot. Our main criterion for selecting parameters of a given method is overall ground truth similarity Sim_B. Note that GraphScope does not accept any user-specified parameters, and thus we leave it out from consideration in this section.

Multi-Step. We test the effect on the method's performance of the similarity threshold parameter θ, which determines when to stop the segment merging process (Supplementary Section S2.2.1). We find that there is no θ value that works well for all of the synthetic network configurations with respect to Sim_B (Supplementary Figure S6a). This is mainly because no single θ value can reliably estimate the ground truth number of segments across the different configurations (Supplementary Figure S6b). Thus, Multi-Step can be used to reliably solve only the CSCD problem where the number of segments is provided as input. So, when comparing Multi-Step against other methods in the context of the SCD problem, we instead ask Multi-Step to solve the CSCD problem with the ground truth number of segments given as input. We refer to this modification of Multi-Step as Multi-Step*. This gives Multi-Step an unfair advantage compared to the other methods, but we have to do this in order to include Multi-Step into comparison.

GHRG. We test the effect on the method's performance of windows size w (Supplementary Section S2.2.1). After varying its values, we observe that the value $w = 4$ generally leads to the highest Sim_B (Supplementary Figure S7). Thus, we use $w = 4$ for our experiments.

SCOUT. We test the effect on the method's performance of a) the objective function, b) consensus clustering method, and c) search strategy.

a) Objective function is used twice (Supplementary Section S2.1.1): 1) when computing the best solution for each possible number of segments (Equation 9), and 2) when choosing among these best solutions the final one (Equation 10). The objective functions can differ between the two cases: in case "1", we can use any Q_P or any Q_B, and in case "2", we have to use a Q_B (Supplementary Section S2.1.1). Thus, since SCOUT uses Q_B up to two times, while it uses Q_P up to one time, we first test the effect of Q_B. Recall that we evaluate two Q_B measures: one based on BIC and the other based on AIC (Supplementary Section S2.1.1). In general, BIC results in higher Sim_B compared to AIC (Supplementary Figure S8a). The reason for this is that AIC produces more segments than BIC, usually overestimating the ground truth number of segments (Supplementary Figure S8b). Recall that this behavior of AIC is not surprising (Supplementary Section S2.1.1). So, we focus on Q_B based on BIC. This gives us the choice of Q_B for case "2". For case "1", we can use Q_B based on BIC or one of the four Q_Ps (based on modularity, conductance, normalized cut, or average-ODF; Supplementary Section S2.1.1). Hence, we next test the effect of Q_P versus Q_B in case "1". Out of all Q_Ps, modularity generally leads to the highest Sim_B (Supplementary Figure S9). However, the best results in terms of Sim_B are achieved when using Q_B based on BIC and not Q_P based on modularity (Supplementary Figure S9). So, whether we are considering case "1" or case "2", Q_B based on BIC overall outperforms all other tested objective functions. Thus, we focus on Q_B based on BIC as SCOUT's objective function.

b) Consensus clustering is used to produce segment partitions, given a segmentation. We use three general types of consensus clustering approaches: sum graph, Average-Louvain, and consensus matrix (Supplementary Section S2.1.2). Recall that the sum graph and consensus matrix approaches are parameterized with the static clustering method (Supplementary Section S2.1.2). So, before we compare the above three general types of approaches, we first aim to choose the best static clustering method for sum graph and consensus matrix approaches. We evaluate seven static clustering methods: Fast Modularity, Label Propagation, Leading Eigenvector, Infomap, Walktrap, Louvain, and Stabilized Louvain (Supplementary Section S2.1.2). We find that generally Walktrap works the best in terms of Sim_B while having comparable running time (Supplementary Figure S10). So, we focus on Walktrap as the static clustering method for both sum graph and consensus matrix approaches. Next, we compare the three general approach types. In terms of Sim_B, all three approaches lead to comparable results (Supplementary Figure S11a), even though Average-Louvain and consensus matrix are more sophisticated compared to sum graph and thus would be expected to be superior. In terms of the running time, sum graph is the fastest of the three approaches (Supplementary Figure S11b). Thus, we focus on sum graph with Walktrap as SCOUT's consensus clustering approach.

c) Search strategy is used to determine how SCOUT searches through the space of possible segmentations. We evaluate three strategies: the exhaustive search, top-down search, and bottom-up search (Supplementary Section S2.1.3). In terms of Sim_B, all three strategies lead to comparable results (Supplementary Figure S12a). In terms of the running time, bottom-up search is the fastest one (it is an order of magnitude faster than the exhaustive search and somewhat faster than the top-down search; Supplementary Figure S12b). Thus, we focus on the bottom-up search as SCOUT's search strategy.

To summarize, we choose Q_B based on BIC as the objective function, sum graph with Walktrap as the consensus clustering method, and the bottom-up search as the search strategy.

Supplementary Algorithms

Supplementary Algorithm S1 SCOUT overview. It has three main components: objective function (**ObjectiveFunction**), consensus clustering (**ConsensusClustering**), and search strategy (**SearchStrategy**). Auxiliary procedure *GetSegmentation* constructs the segmentation of a dynamic network given a change point set and auxiliary procedure *GetBestOutput* returns the best (with respect to the objective function) of all considered outputs.

Input: D
Output: $A^\star = (T^\star, P^\star)$

$R \leftarrow \emptyset$
$T \leftarrow$ **SearchStrategy**$(\varnothing, R)$
while $T \neq \varnothing$ **do**
  $S \leftarrow \mathit{GetSegmentation}(T, D)$
  $P \leftarrow \emptyset$
  for $s \in S$ **do**
    $P \leftarrow P \cup \{$**ConsensusClustering**$(s)\}$
  end for
  $A \leftarrow (T, P)$
  $R \leftarrow R \cup \{(A,$ **ObjectiveFunction**$(A))\}$
  $T \leftarrow$ **SearchStrategy**(T, R)
end while
return $\mathit{GetBestOutput}(R)$

Supplementary Algorithm S2 Step 3 of our synthetic network generation process. Auxiliary procedure *RandomPartition* randomly partitions a given set into a given number of clusters and auxiliary procedure *Outneighbors* returns the list of the outneighbors of a given supernode.

Input: G_P
Output: $P^{(gt)}$

$V \leftarrow [0, n-1]$
$p_0^{(gt)} \leftarrow RandomPartition(V, |V_P^{(0)}|)$
for $i \in [1, l-1]$ **do**
  for $v \in V_P^{(i)}$ **do**
    $c_{(v)} \leftarrow \emptyset$
  end for
  for $u \in V_P^{(i-1)}$ **do**
    $p^{(temp)} \leftarrow RandomPartition(c_{(u)}, d^+(u))$
    $j \leftarrow 0$
    for $c^{(temp)} \in p^{(temp)}$ **do**
      $c_{(Outneighbors(u)[j])} \leftarrow c_{(Outneighbors(u)[j])} \cup p^{(temp)}$
      $j \leftarrow j + 1$
    end for
  end for
  $p_i^{(gt)} \leftarrow \{c_{(v)} | v \in V_P^{(i)}\}$
end for
$P^{(gt)} \leftarrow \{p_i^{(gt)} | i \in [0, l-1]\}$
return $P^{(gt)}$

Supplementary Figures

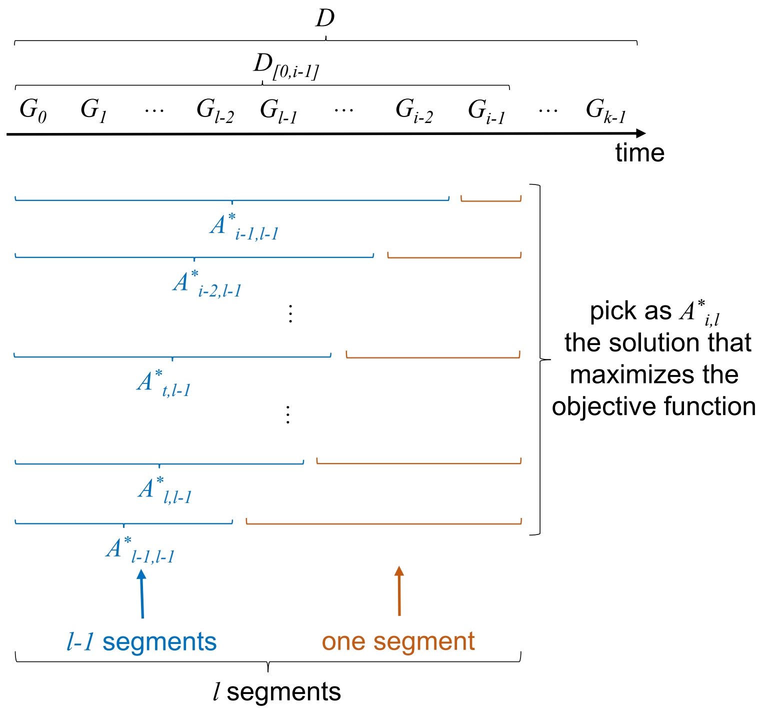

Supplementary Figure S1: The procedure of obtaining solution $A^{\star}_{i,l}$ for $D_{[0,i-1]}$ that has l segments during the ith iteration of the exhaustive search. For this, we construct a set of candidate solutions (shown in rows) and pick as $A^{\star}_{i,l}$ the one solution from this set that maximizes the objective function. Each candidate solution is obtained by combining: 1) solution $A^{\star}_{t,l-1}$ that has $l-1$ segments, which was obtained in one of the previous iterations (shown in blue), and 2) a solution with one segment, whose only segment partition is obtained during the current iteration (shown in orange).

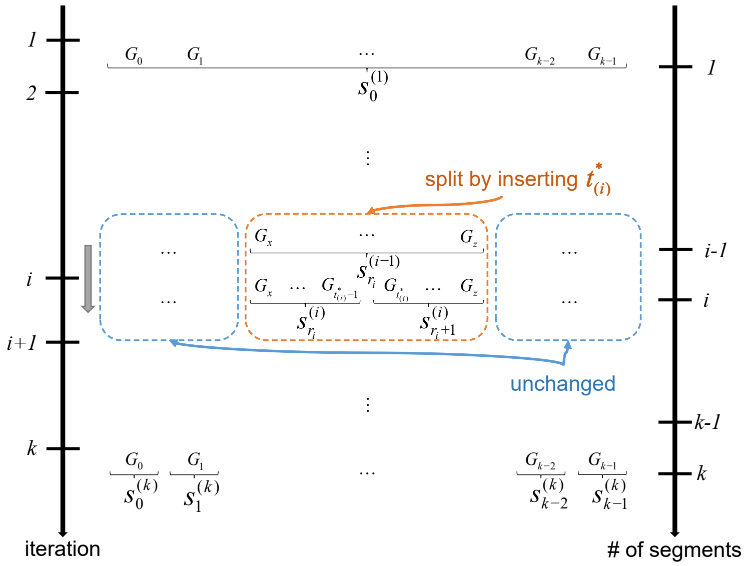

Supplementary Figure S2: The top-down search. The procedure starts with one segment encompassing the whole network and then iteratively splits one of the current segments into two. For the ith iteration, the orange box indicates the segment that was split as a result of this iteration, and blue boxes indicate all other, unchanged segments.

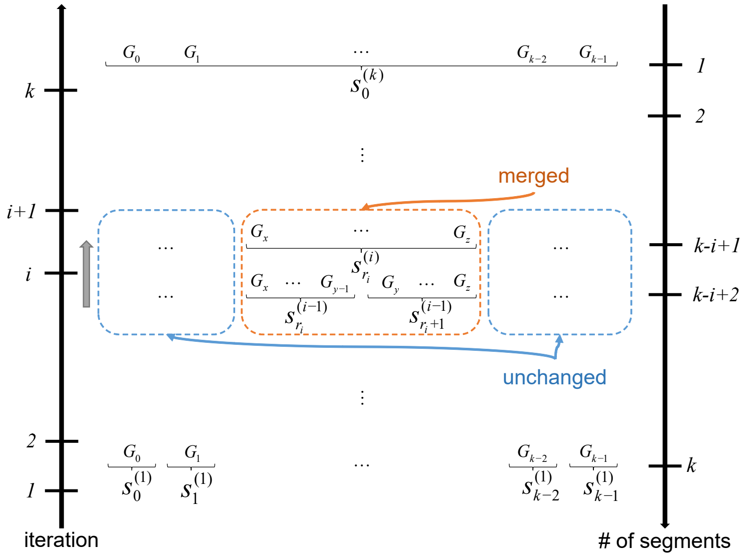

Supplementary Figure S3: The bottom-up search. The procedure starts with k singleton segments and then iteratively merges two of the current adjacent segments into one. For the ith iteration, the orange box indicates the segment that was created as a result of this iteration, and blue boxes indicate all other, unchanged segments.

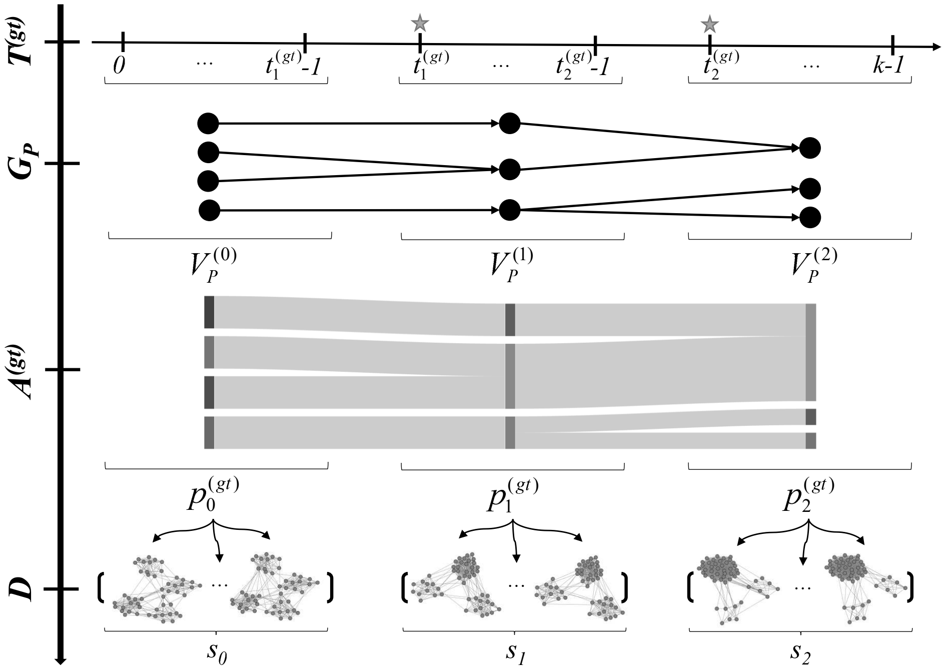

Supplementary Figure S4: The process of constructing synthetic dynamic network D with known ground truth solution $A^{(gt)} = (T^{(gt)}, P^{(gt)})$. The four steps of the process, described in the text, are illustrated from top to bottom.

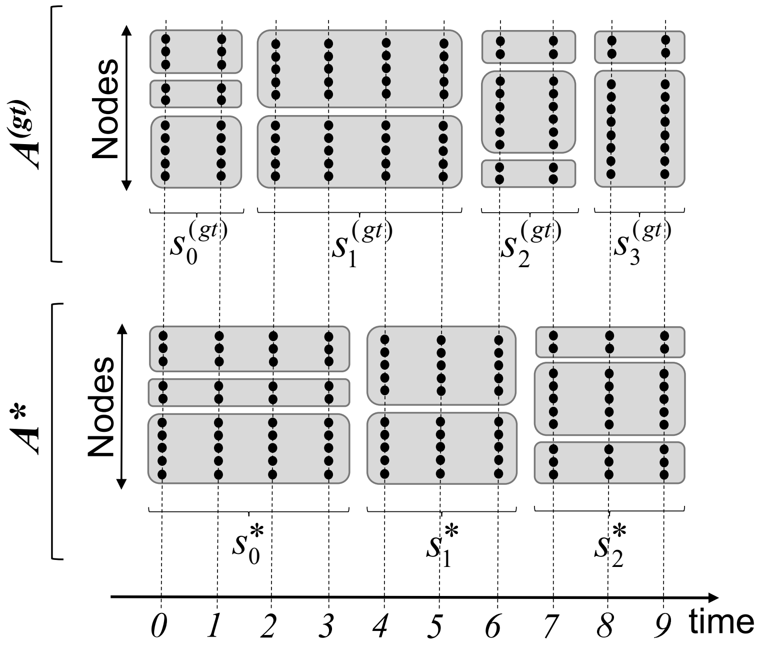

Supplementary Figure S5: The process of constructing a node-time partition $\mathcal{P}_B$ when computing Sim_B. Each black circle corresponds to a node at a given time point. Circles on the same horizontal line correspond to a fixed node at different time points. Circles on the same vertical line correspond to different nodes at a fixed time point. Rectangles illustrate clusters in node-time partitions $\mathcal{P}^{\star}_B$ and $\mathcal{P}^{(gt)}_B$.

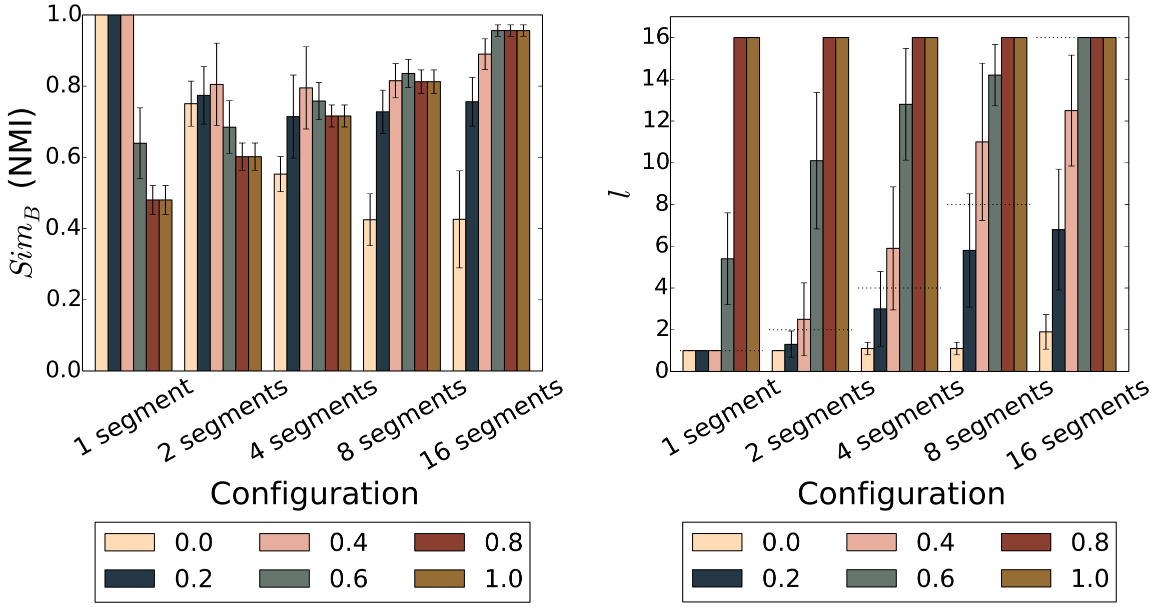

(a) (b)

Supplementary Figure S6: The effect of θ value for Multi-Step, in terms of **(a)** Sim_B and **(b)** the number of segments l, as we vary the ground truth synthetic network configuration (x-axis). Note that we run the analysis for $\theta \in [0, 2]$ in increments of 0.1. However, since the results for $\theta \geq 1.0$ are all the same, we show the results only for $\theta \leq 1.0$, and for visual clarity, we show the results in increments of 0.2. In panel (b), for a given ground truth configuration, the dotted line corresponds to the ground truth number of segments.

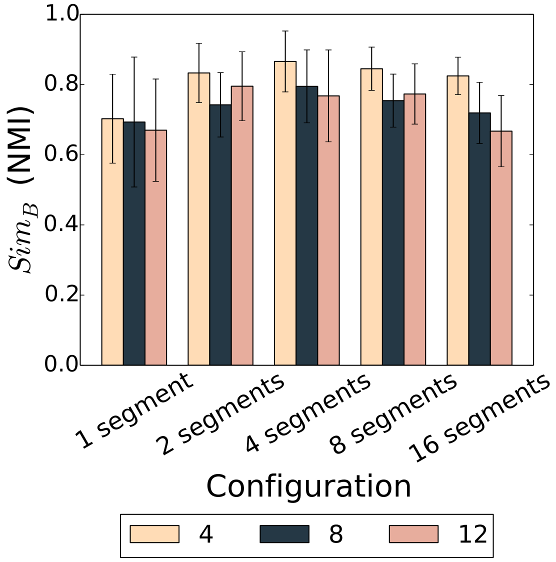

Supplementary Figure S7: The effect of w value for GHRG, in terms of Sim_B, as we vary the ground truth synthetic network configuration (x-axis).

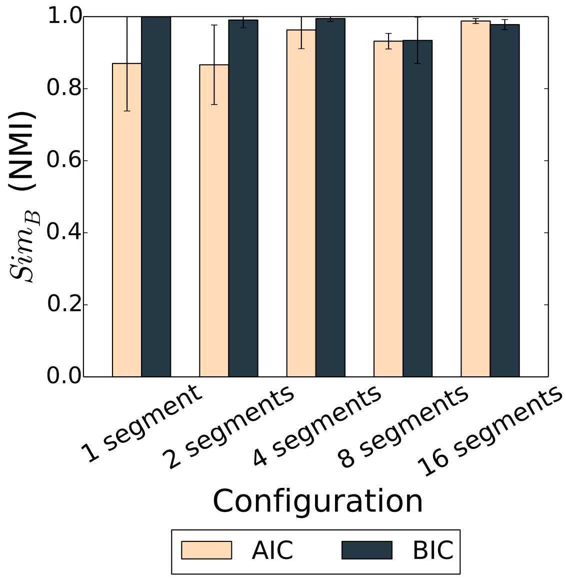

(a)

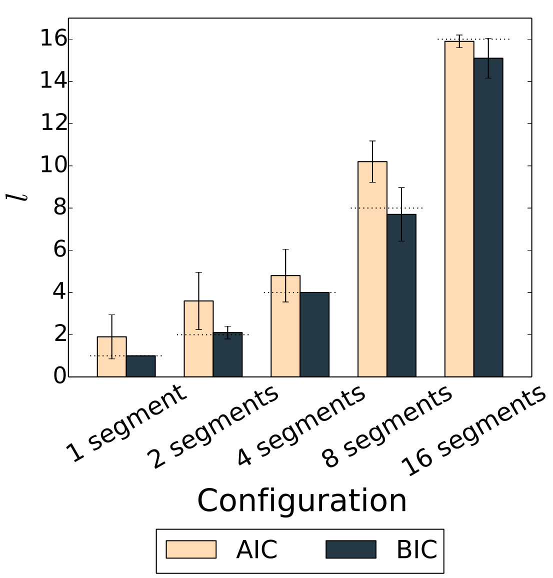

(b)

Supplementary Figure S8: The effect of Q_B choice for SCOUT when choosing the optimal number of segments, in terms of **(a)** Sim_B and **(b)** the number of segments l, as we vary the ground truth synthetic network configuration (x-axis). Here, in all cases, we fix consensus clustering method as sum graph with Walktrap and search strategy as exhaustive search. In panel (b), for a given ground truth configuration, the dotted line corresponds to the ground truth number of segments.

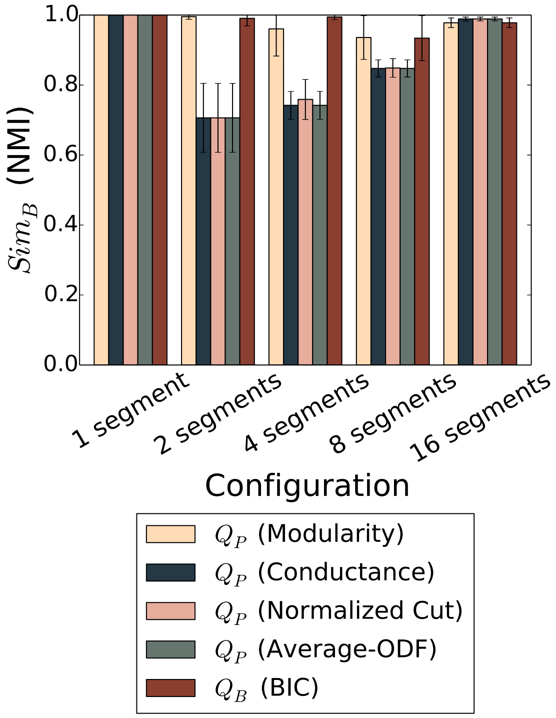

Supplementary Figure S9: The effect of Q choice for SCOUT when producing the best solution for each possible number of segments, in terms of Sim_B, as we vary the ground truth synthetic network configuration (x-axis). Note that when using Q_P (the first four series), we still need to choose the optimal number of segments, which we do using Q_B (i.e., BIC). Here, in all cases, we fix consensus clustering method as sum graph with Walktrap, and we fix search strategy as exhaustive search.

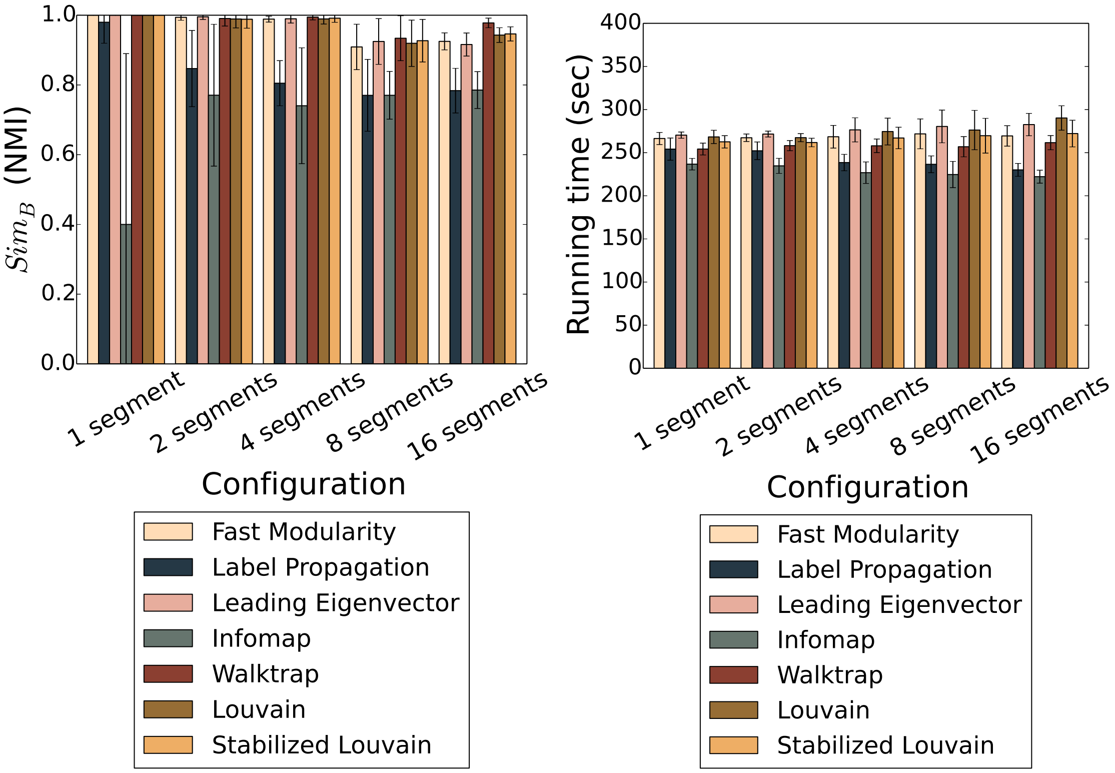

(a) (b)

Supplementary Figure S10: The effect of the choice of static clustering method in the sum graph consensus clustering method for SCOUT, in terms of **(a)** Sim_B and **(b)** running time, as we vary the ground truth synthetic network configuration (x-axis). Here, in all cases, we fix search strategy as exhaustive search.

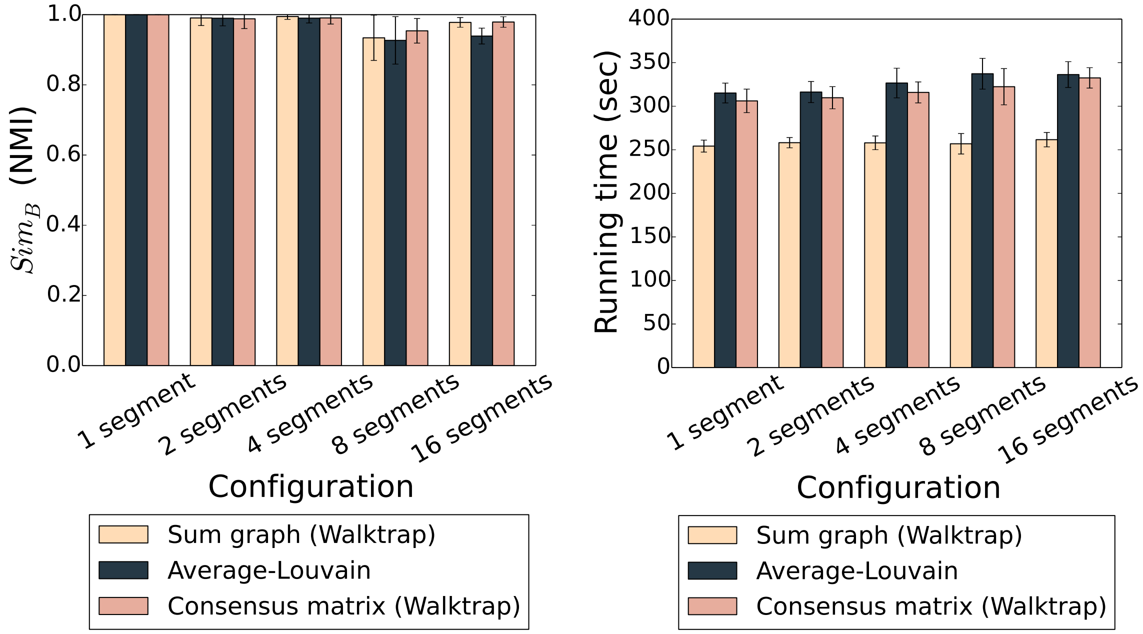

(a) (b)

Supplementary Figure S11: The effect of the choice of consensus clustering method for SCOUT, in terms of **(a)** Sim_B and **(b)** running time, as we vary the ground truth synthetic network configuration (x-axis). Here, in all cases, we fix search strategy as exhaustive search.

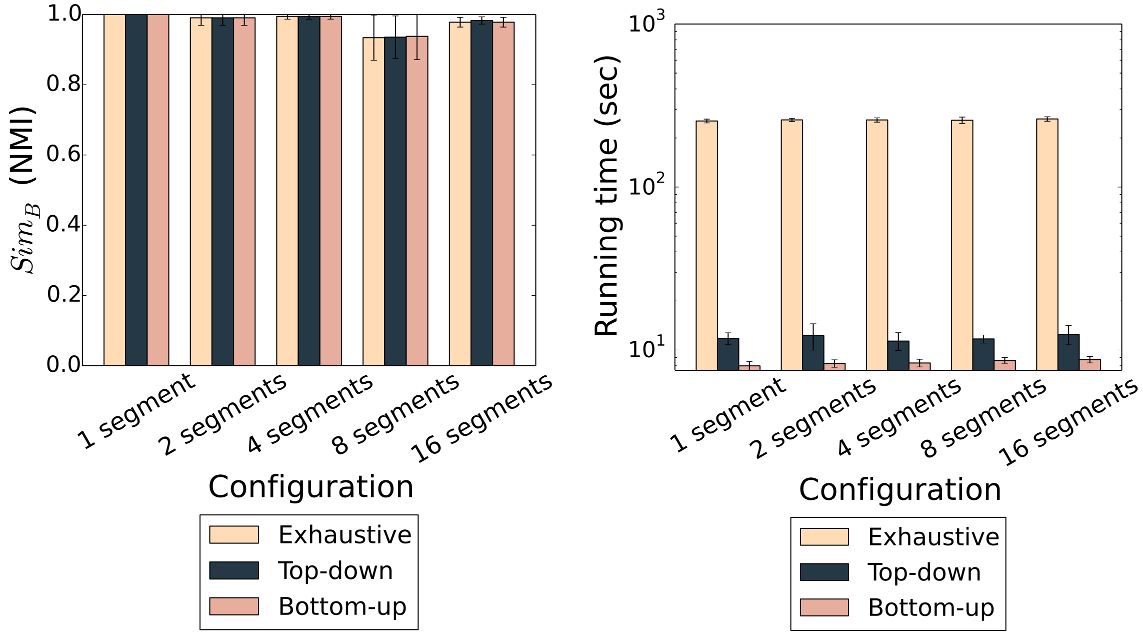

(a) (b)

Supplementary Figure S12: The effect of the choice of search strategy for SCOUT, in terms of **(a)** Sim_B and **(b)** running time (logarithmic scale), as we vary the ground truth synthetic network configuration (x-axis).

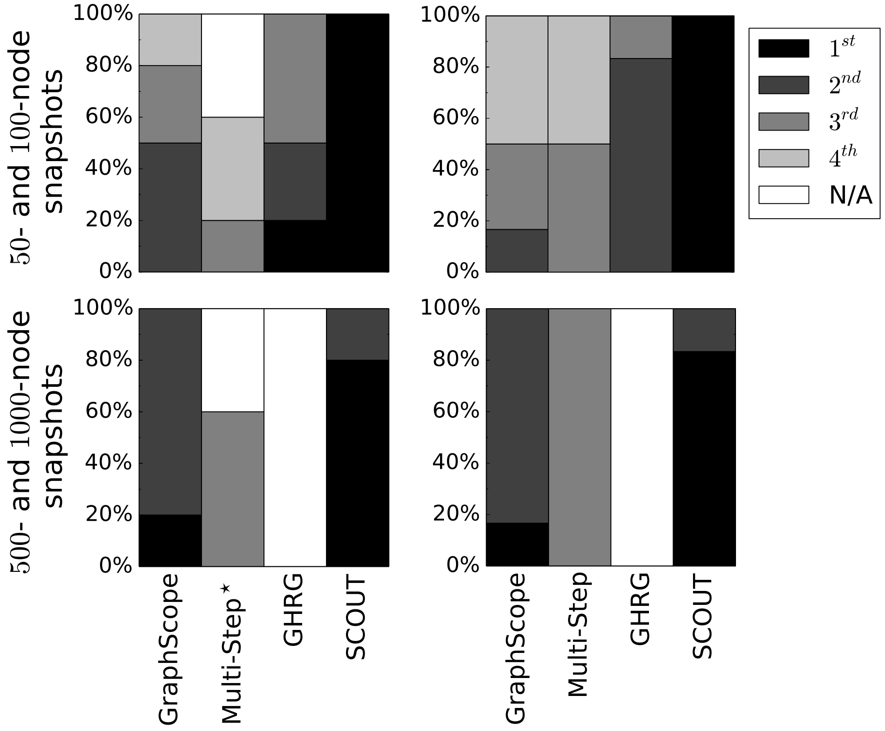

(a) Sim_T

(b) Change point classification

Supplementary Figure S13: Rankings of the methods with respect to **(a)** Sim_T and **(b)** change point classification. Since GHRG could not be run for the larger networks, the results are split into those for the configurations with 50 and 100 nodes per snapshot (top) and those for the configurations with 500 and 1000 nodes per snapshot (bottom). Note that for change point classification, we exclude from consideration configurations with the minimum (i.e., one) and maximum (i.e., 16) possible numbers of ground truth segments. This is because for these configurations, either there are no change points at all (i.e., for one segment) or every time point is a change point (i.e., for 16 segments), so change point classification cannot be performed. The rankings are computed as follows. For each synthetic network configuration, we compare the four methods' scores (average scores over all instances of the given configuration) to identify the first, second, third, and fourth best method; ties are allowed, in which case, two methods would be assigned the same rank. Then, we summarize these results over all considered synthetic network configurations by measuring, for each method (x-axis), how many times the given method is ranked as the first, second, third, and fourth best method (expressed as the percentage of all considered configurations; y-axis). "N/A" indicates that either a given method could not be run (which is the case for GHRG for the larger networks in both panels) or it was excluded from the consideration (which is the case for Multi-Step in panel (a)). The reason we exclude Multi-Step from the consideration in certain configurations in panel (a) (namely, those with the minimum and maximum possible numbers of segments) is as follows. Since we provide Multi-Step with the ground truth number of segments as input and since there is only one possible segmentation with minimum or maximum possible number of segments, Multi-Step trivially returns the perfect Sim_T score for these extreme configurations. The figure can intuitively be interpreted as follows: the darker the bar of a given method, the better its performance.

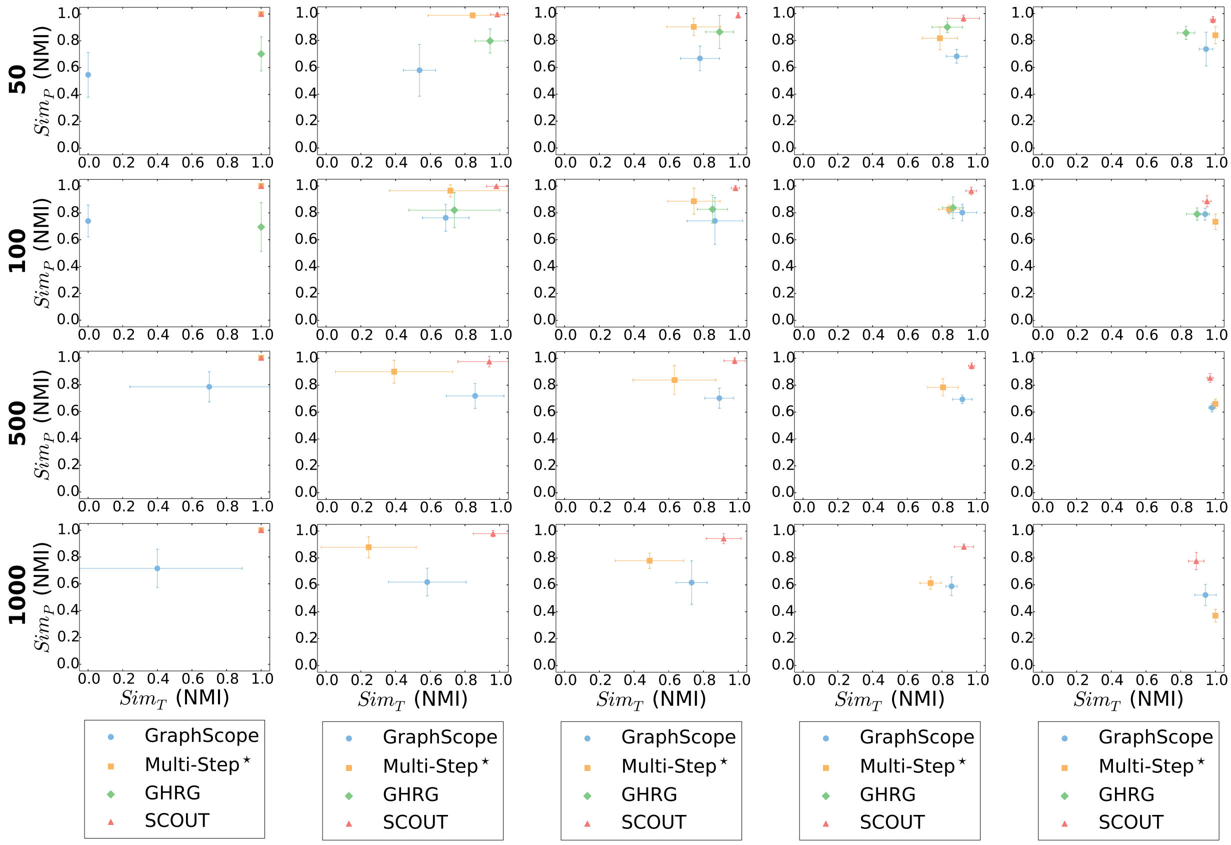

(a) 1 segment (b) 2 segments (c) 4 segments (d) 8 segments (e) 16 segments

Supplementary Figure S14: Sim_T and Sim_P scores for all 20 synthetic network configurations. The rows correspond to four different numbers of nodes per snapshot, with the number of nodes increasing from top to bottom. The columns correspond to five different numbers of ground truth segments: **(a)** one ground truth segment, **(b)** two ground truth segments, **(c)** four ground truth segments, **(d)** eight ground truth segments, and **(e)** 16 ground truth segments. In each panel, for each method, the results are averaged over all of the corresponding synthetic network instances.

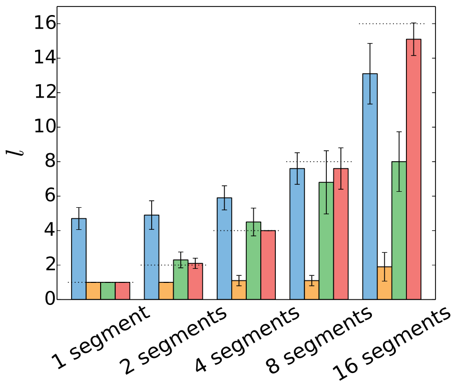

(a) 50-node snapshots

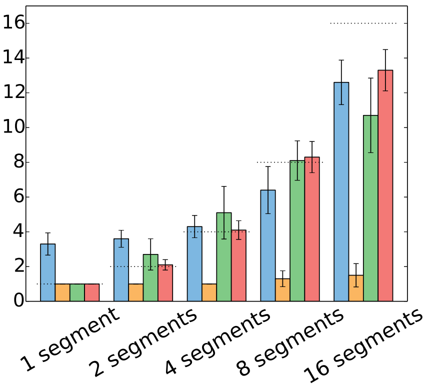

(b) 100-node snapshots

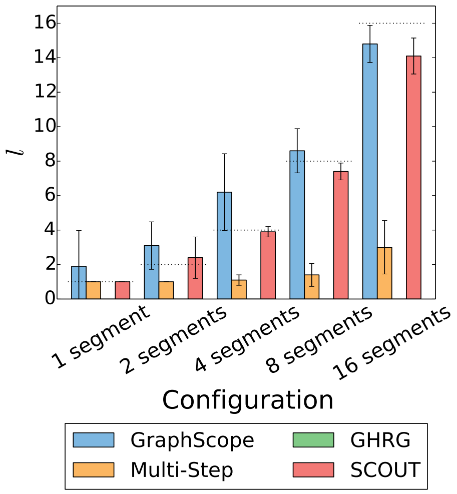

(c) 500-node snapshots

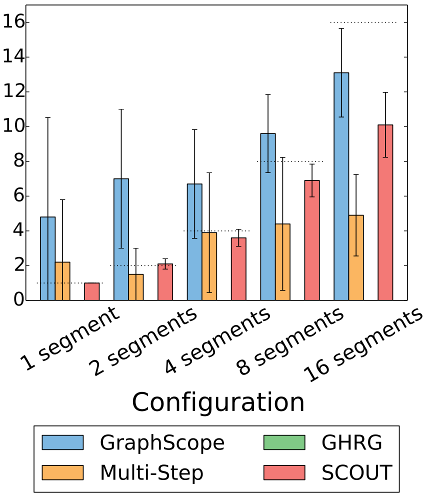

(d) 1000-node snapshots

Supplementary Figure S15: The number of segments l in the solutions produced by the methods for synthetic networks with **(a)** 50-node snapshots, **(b)** 100-node snapshots, **(c)** 500-node snapshots, and **(d)** 1000-node snapshots. In each panel, the results are grouped by the number of ground truth segments and averaged over all of the corresponding synthetic network instances. A given dotted line corresponds to the ground truth number of segments in the given synthetic network configuration. Note that for Multi-Step, we use the default parameters, since when using Multi-Step* that is provided with the number of ground truth segments as input, this method trivially returns the correct number of ground truth segments.

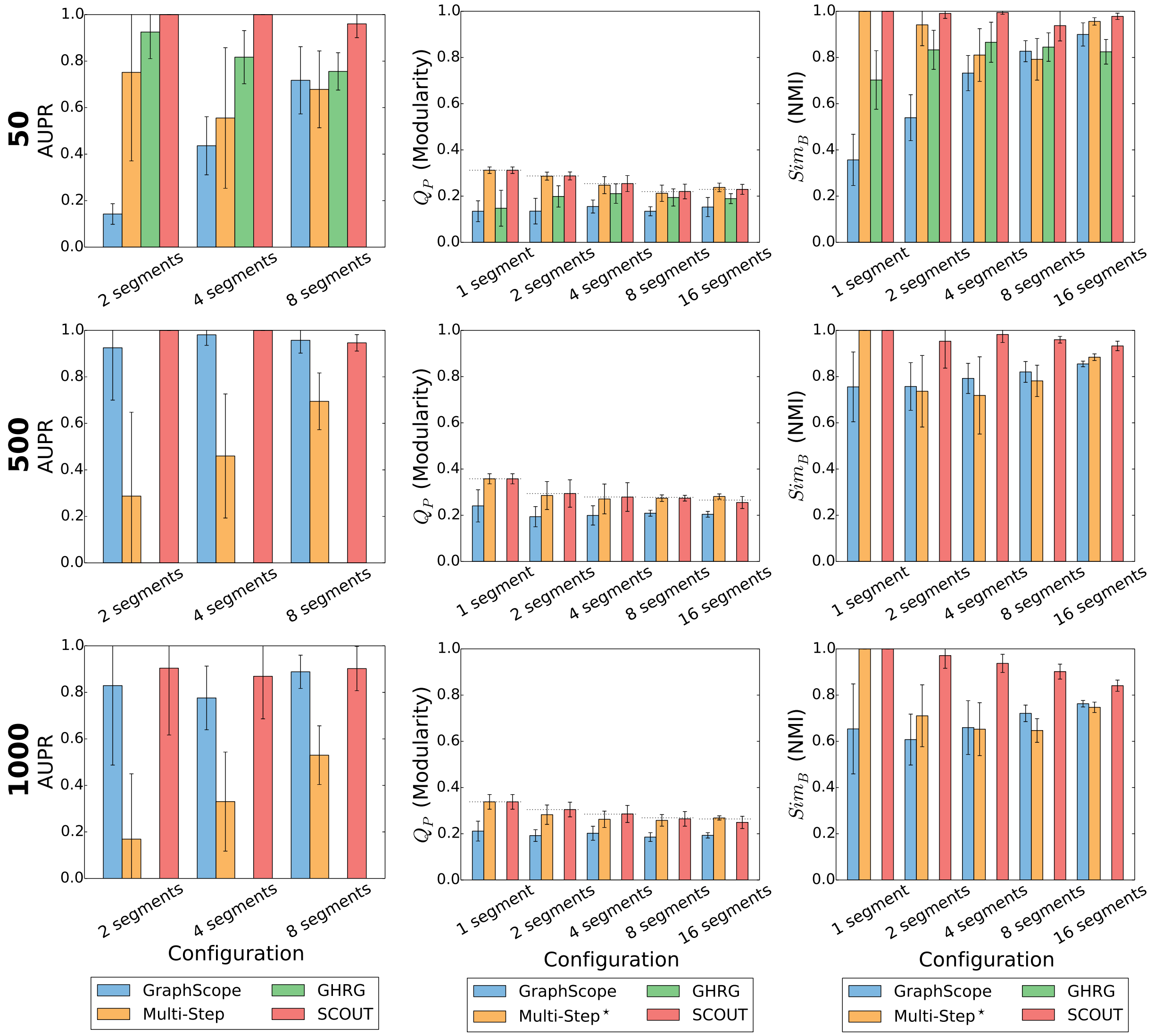

(a) Change point classification

(b) Partition quality Q_P

(c) Overall similarity Sim_B

Supplementary Figure S16: Method comparison for synthetic networks with 50, 500, and 1000 nodes per snapshot (shown from top to bottom) with respect to **(a)** change point classification, **(b)** Q_P, and **(c)** Sim_B. For a given ground truth configuration, the results are averaged over all of the corresponding synthetic network instances. In panel (b), the dotted lines correspond to the ground truth score. Note that for panel (a), we exclude from consideration the configurations with the minimum and maximum possible numbers of ground truth segments. We do this because for these configurations, either there are no change points at all (for one segment) or every time point is a change point (for 16 segments), which means that change point classification cannot be performed. GHRG could not be run for two largest network sizes due to its high computational complexity.

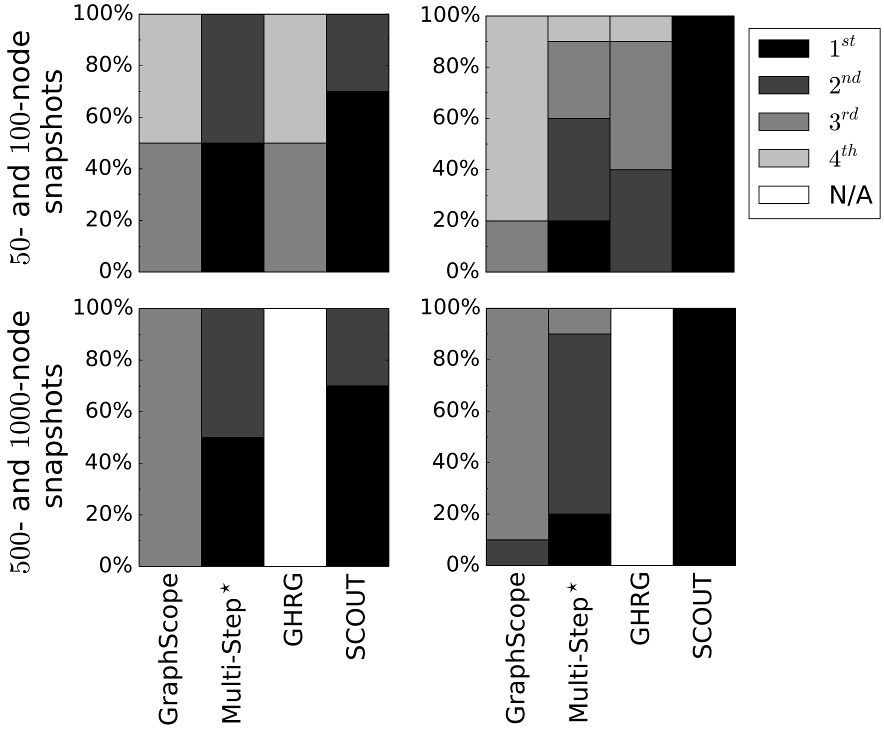

(a) Q_P

(b) Sim_P

Supplementary Figure S17: Rankings of the methods with respect to **(a)** Q_P and **(b)** Sim_P. Since GHRG could not be run for the larger networks, the results are split into those for the configurations with 50 and 100 nodes per snapshot (top) and those for the configurations with 500 and 1000 nodes per snapshot (bottom). The rankings are computed as follows. For each synthetic network configuration, we compare the four methods' scores (average scores over all instances of the given configuration) to identify the first, second, third, and fourth best method; ties are allowed, in which case, two methods would be assigned the same rank. Then, we summarize these results over all considered synthetic network configurations by measuring, for each method (x-axis), how many times the given method is ranked as the first, second, third, and fourth best method (expressed as the percentage of all considered configurations; y-axis). "N/A" indicates that the given method could not be run (which is the case for GHRG for the larger networks). The figure can intuitively be interpreted as follows: the darker the bar of a given method, the better its performance.

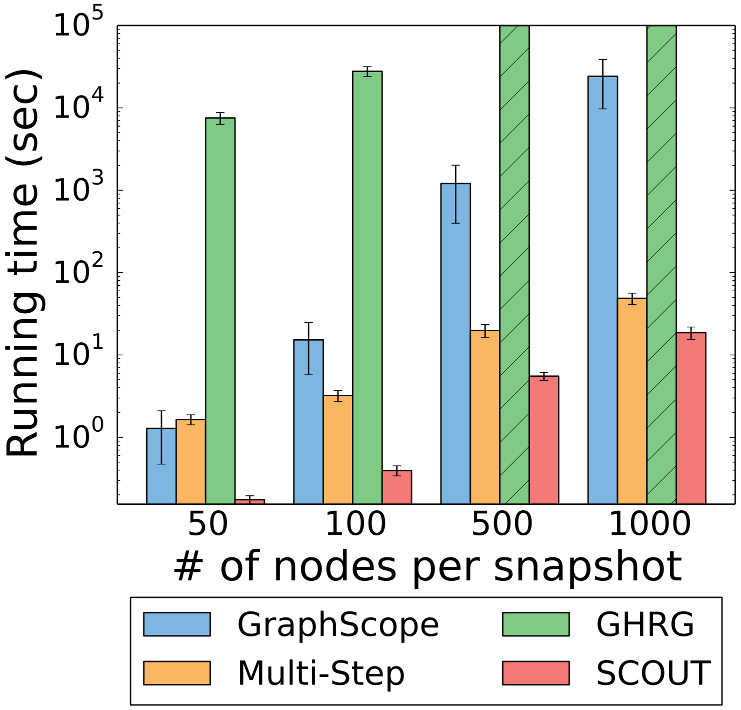

Supplementary Figure S18: Running times of the methods for synthetic networks (logarithmic scale). The results are grouped by the number of nodes per snapshot. For each number of nodes, running times are averaged over the corresponding numbers of segments and synthetic network instances (i.e., over $5 \times 10 = 50$ runs). The striped bars mean that the method could not finish within the allowed time.

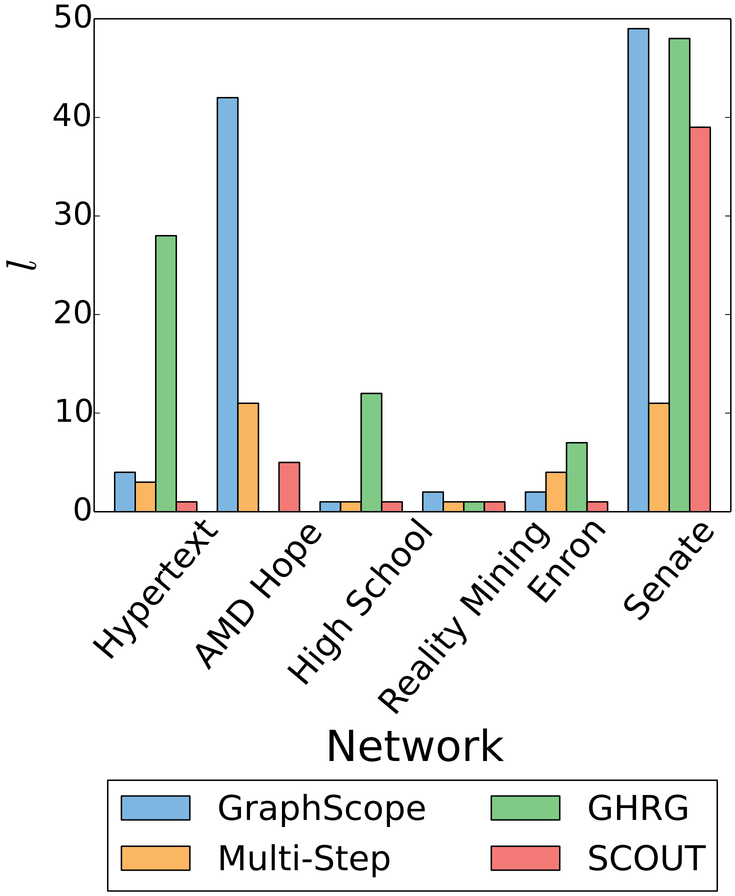

Supplementary Figure S19: The number of segments l in the solutions produced by the methods for real-world networks.

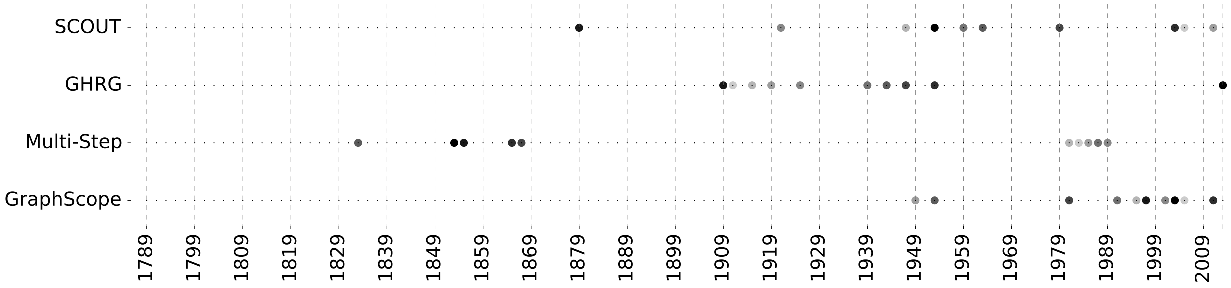

Supplementary Figure S20: The top 10 highest ranked time points by each method for *Senate* network. Time points (expressed in years) are shown on the x-axis. Methods are shown on the y-axis. Each circle corresponds to one of the top 10 ranked time points, and the darker its color, the higher its rank.

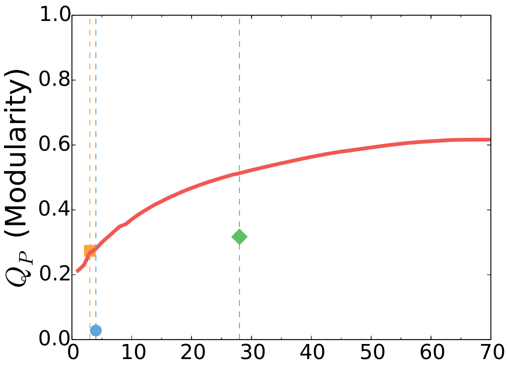

(a) *Hypertext*

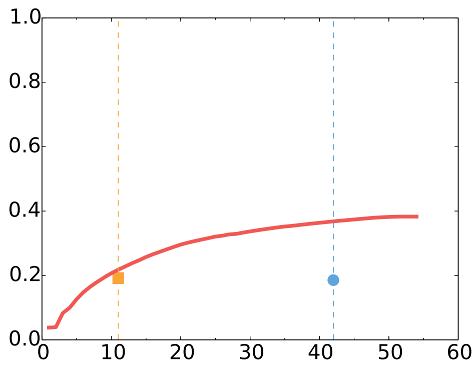

(b) *AMD Hope*

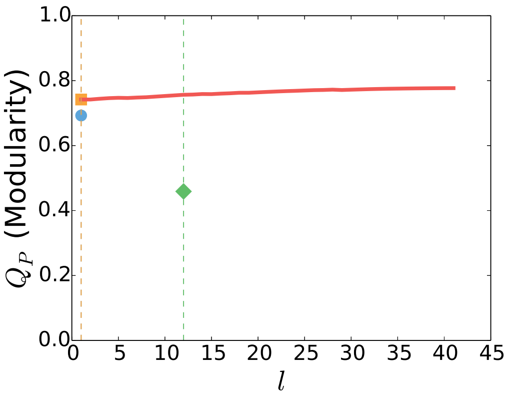

(c) *High School*

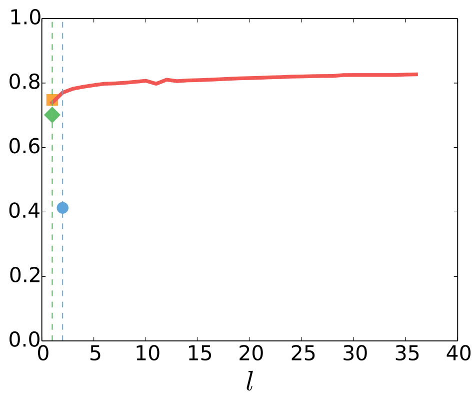

(d) *Reality mining*

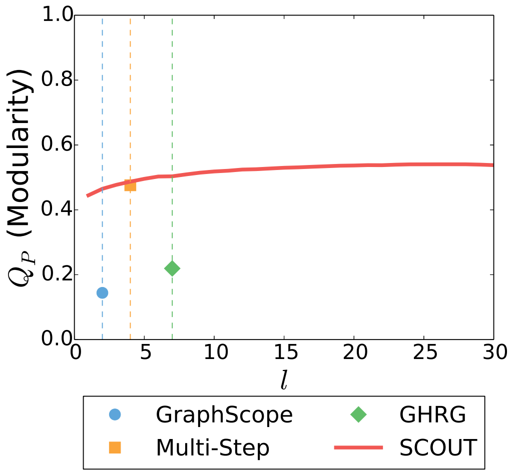

(e) *Enron*

Supplementary Figure S21: Q_P scores of 1) SCOUT's solutions for different numbers of segments l and 2) the solutions of the existing methods for **(a)** *Hypertext* network, **(b)** *AMD Hope* network, **(c)** *High School* network, **(d)** *Reality Mining* network, and **(e)** *Enron* network. For SCOUT, the line shows its Q_P score when solving the CSCD problem while varying the number of segments. For each of the existing methods, the mark shows Q_P score of its solution, with the position of the mark along the x-axis corresponding to the number of segments l in the solution.

Supplementary Tables

Supplementary Table S1: Statistical significance of the superiority of SCOUT over the best existing method in terms of Sim_B on synthetic networks. "Configuration" columns describe the synthetic network configurations in terms of the number of nodes per snapshot and the number of segments. "Average Sim_B" columns contain Sim_B scores of SCOUT and the best of all existing methods (shown in the parentheses), where the scores are averaged over all corresponding synthetic network instances; the highest Sim_B score for a given configuration (i.e., in the given row) is shown in bold. The "p-value" column shows the statistical significance of the difference between Sim_B scores of SCOUT and the best existing method. "N/A" means that the scores are identical. p-values less than 0.05 are shown in bold, p-values less than 0.01 are shown with one star, and p-values less than 0.001 are shown with two stars.

Configuration		Average Sim_B		p-value
# of nodes	# of segments	SCOUT	Best existing method	
50	1	**1.000**	**1.000** (Multi-Step⋆)	N/A
	2	**0.991**	0.941 (Multi-Step⋆)	1.548E-01
	4	**0.995**	0.866 (GHRG)	**2.075E-03**⋆
	8	**0.938**	0.845 (GHRG)	**4.889E-04**⋆⋆
	16	**0.978**	0.956 (Multi-Step⋆)	**3.876E-04**⋆⋆
100	1	**1.000**	**1.000** (Multi-Step⋆)	N/A
	2	**0.989**	0.877 (Multi-Step⋆)	**4.643E-02**
	4	**0.986**	0.818 (GHRG)	**4.045E-04**⋆⋆
	8	**0.966**	0.872 (GraphScope)	**1.691E-03**⋆
	16	**0.931**	0.918 (Multi-Step⋆)	**3.311E-02**
500	1	**1.000**	**1.000** (Multi-Step⋆)	N/A
	2	**0.953**	0.757 (GraphScope)	**1.408E-05**⋆⋆
	4	**0.982**	0.792 (GraphScope)	**8.709E-06**⋆⋆
	8	**0.960**	0.820 (GraphScope)	**2.047E-05**⋆⋆
	16	**0.933**	0.884 (Multi-Step⋆)	**1.628E-05**⋆⋆
1000	1	**1.000**	**1.000** (Multi-Step⋆)	N/A
	2	**0.971**	0.710 (Multi-Step⋆)	**4.666E-04**⋆⋆
	4	**0.937**	0.659 (GraphScope)	**1.677E-04**⋆⋆
	8	**0.902**	0.721 (GraphScope)	**3.058E-06**⋆⋆
	16	**0.841**	0.763 (GraphScope)	**6.030E-06**⋆⋆

Supplementary Table S2: Real-world networks that we use in our study. "Network" columns show general information about a given network. "Snapshots" columns show properties of the network snapshots averaged over all snapshots.

	Network				Snapshots		
Name	# of nodes	# of edges	Time span	Edge type	# of nodes	# of edges	Duration
Hypertext	113	21K	3 days	Proximity	47 (20)	73 (53)	30 min
AMD Hope	409	1.26M	3 days	Co-location	161 (101)	4.5K (3.9K)	1 hour
High School	327	189K	5 days	Proximity	227 (39)	499 (237)	1 hour
Reality Mining	78	5K	10 months	Phone call	28 (11)	25 (12)	1 week
Enron	184	121K	2.5 years	Email	99 (40)	267 (154)	1 month
Senate	51	28K	227 years	Voting similarity	35 (15)	248 (268)	2 years

References

[1] J. Sun, C. Faloutsos, S. Papadimitriou, and P. S. Yu, “GraphScope: parameter-free mining of large time-evolving graphs,” in *Proceedings of the 13th ACM SIGKDD International Conference on Knowledge Discovery and Data Mining.* ACM, 2007, pp. 687–696.

[2] J. Ferlez, C. Faloutsos, J. Leskovec, D. Mladenic, and M. Grobelnik, “Monitoring network evolution using MDL,” in *Data Engineering, 2008. ICDE 2008. IEEE 24th International Conference on.* IEEE, 2008, pp. 1328–1330.

[3] D. Duan, Y. Li, Y. Jin, and Z. Lu, “Community mining on dynamic weighted directed graphs,” in *Proceedings of the 1st ACM International Workshop on Complex Networks Meet Information & Knowledge Management.* ACM, 2009, pp. 11–18.

[4] T. Aynaud and J.-L. Guillaume, “Multi-step community detection and hierarchical time segmentation in evolving networks,” in *Proceedings of the 5th SNA-KDD workshop*, 2011.

[5] L. Peel and A. Clauset, “Detecting change points in the large-scale structure of evolving networks,” in *Twenty-Ninth AAAI Conference on Artificial Intelligence*, 2015.

[6] J. Yang and J. Leskovec, “Defining and evaluating network communities based on ground-truth,” *Knowledge and Information Systems*, vol. 42, no. 1, pp. 181–213, 2015.

[7] M. E. Newman and M. Girvan, “Finding and evaluating community structure in networks,” *Physical Review E*, vol. 69, no. 2, p. 026113, 2004.

[8] X.-Q. Cheng and H.-W. Shen, “Uncovering the community structure associated with the diffusion dynamics on networks,” *Journal of Statistical Mechanics: Theory and Experiment*, vol. 2010, no. 04, p. P04024, 2010.

[9] J. Shi and J. Malik, “Normalized cuts and image segmentation,” *Pattern Analysis and Machine Intelligence, IEEE Transactions on*, vol. 22, no. 8, pp. 888–905, 2000.

[10] G. W. Flake, S. Lawrence, and C. L. Giles, “Efficient identification of web communities,” in *Proceedings of the Sixth ACM SIGKDD International Conference on Knowledge Discovery and Data Mining.* ACM, 2000, pp. 150–160.

[11] J. B. Kadane and N. A. Lazar, “Methods and criteria for model selection,” *Journal of the American Statistical Association*, vol. 99, no. 465, pp. 279–290, 2004.

[12] P. Grünwald, “Model selection based on minimum description length,” *Journal of Mathematical Psychology*, vol. 44, no. 1, pp. 133–152, 2000.

[13] H. Akaike, “Information theory and an extension of the maximum likelihood principle,” in *Selected Papers of Hirotugu Akaike.* Springer, 1998, pp. 199–213.

[14] G. Schwarz *et al.*, “Estimating the dimension of a model,” *The Annals of Statistics*, vol. 6, no. 2, pp. 461–464, 1978.

[15] B. Karrer and M. E. Newman, “Stochastic blockmodels and community structure in networks,” *Physical Review E*, vol. 83, no. 1, p. 016107, 2011.

[16] A. Lancichinetti and S. Fortunato, “Consensus clustering in complex networks,” *Scientific Reports*, vol. 2, 2012.

[17] S. Fortunato, “Community detection in graphs,” *Physics Reports*, vol. 486, no. 3, pp. 75–174, 2010.

[18] A. Clauset, M. E. Newman, and C. Moore, “Finding community structure in very large networks,” *Physical Review E*, vol. 70, no. 6, p. 066111, 2004.

[19] U. N. Raghavan, R. Albert, and S. Kumara, “Near linear time algorithm to detect community structures in large-scale networks,” *Physical Review E*, vol. 76, no. 3, p. 036106, 2007.

[20] M. E. Newman, “Finding community structure in networks using the eigenvectors of matrices,” *Physical Review E*, vol. 74, no. 3, p. 036104, 2006.

[21] M. Rosvall, D. Axelsson, and C. T. Bergstrom, “The map equation,” *The European Physical Journal Special Topics*, vol. 178, no. 1, pp. 13–23, 2010.

[22] P. Pons and M. Latapy, “Computing communities in large networks using random walks,” in *Computer and Information Sciences-ISCIS 2005*. Springer, 2005, pp. 284–293.

[23] V. D. Blondel, J.-L. Guillaume, R. Lambiotte, and E. Lefebvre, “Fast unfolding of communities in large networks,” *Journal of Statistical Mechanics: Theory and Experiment*, vol. 2008, no. 10, p. P10008, 2008.

[24] T. Aynaud and J.-L. Guillaume, “Static community detection algorithms for evolving networks,” in *Modeling and Optimization in Mobile, Ad Hoc and Wireless Networks (WiOpt), 2010 Proceedings of the 8th International Symposium on*. IEEE, 2010, pp. 513–519.

[25] S. Beis, “Implementation of GraphScope algorithm for clustering time evolving graphs,” https://github.com/sarovios/social-graph-cluster, 2014.

[26] M. Newman and A. Clauset, “Structure and inference in annotated networks,” *arXiv preprint arXiv:1507.04001*, 2015.

[27] L. Isella, J. Stehlé, A. Barrat, C. Cattuto, J.-F. Pinton, and W. Van den Broeck, “What’s in a crowd? analysis of face-to-face behavioral networks,” *Journal of Theoretical Biology*, vol. 271, no. 1, pp. 166–180, 2011.

[28] “AMD Hope RFID Data,” http://networkdata.ics.uci.edu/data.php?d=amdhope, 2008.

[29] R. Mastrandrea, J. Fournet, and A. Barrat, “Contact patterns in a high school: a comparison between data collected using wearable sensors, contact diaries and friendship surveys,” *PLOS ONE*, vol. 10, no. 9, p. e0136497, 2015.

[30] N. Eagle and A. Pentland, “Reality Mining: Sensing Complex Social Systems,” *Personal and Ubiquitous Computing*, vol. 10, no. 4, pp. 255–268, 2006.

[31] C. E. Priebe, J. M. Conroy, D. J. Marchette, and Y. Park, “Scan statistics on Enron graphs,” *Computational & Mathematical Organization Theory*, vol. 11, no. 3, pp. 229–247, 2005.

[32] V. Kawadia and S. Sreenivasan, “Sequential detection of temporal communities by estrangement confinement,” *Scientific Reports*, vol. 2, 2012.

[33] N. X. Vinh, J. Epps, and J. Bailey, “Information theoretic measures for clusterings comparison: Variants, properties, normalization and correction for chance,” *The Journal of Machine Learning Research*, vol. 11, pp. 2837–2854, 2010.

[34] A. Rosenberg and J. Hirschberg, “V-measure: A conditional entropy-based external cluster evaluation measure.” in *EMNLP-CoNLL*, vol. 7, 2007, pp. 410–420.
